# Supplementary material for: The core microbiomes and associated metabolic potential of water kefir as revealed by pan multi-omics
Source: Commun Biol. 2025 Mar 11;8:415. doi: 10.1038/s42003-025-07808-3 (PMC11897133; doi:10.1038/s42003-025-07808-3)
Supplement: Supplementary file 2 — Supplementary Material [file 42003_2025_7808_MOESM2_ESM.pdf]

The core microbiomes and associated metabolic potential of water kefir as revealed by pan multi-omics

Samuel Breselge[1,2], Iwona Skibinska[1], Xiaofei Yin[3,4], Lorraine Brennan[3,4,5], Kieran Kilcawley[1,6] & Paul D. Cotter[1,2,5,7]

Author Affiliations:

[1]Teagasc Food Research Centre, Moorepark, Cork, Ireland

[2]APC Microbiome Ireland, Cork, Ireland

[3]UCD Institute of Food and Health, UCD School of Agriculture and Food Science, University College Dublin, Dublin, Ireland

[4]UCD Conway Institute of Biomolecular and Biomedical Research, University College Dublin, Dublin, Ireland

[5]VistaMilk, Cork, Ireland

[6]School of Food and Nutritional Sciences, University College Cork, Cork, Ireland

[7]Corresponding author (Paul.Cotter@teagasc.ie)

Supplementary Material and Methods:

Test for freeze tolerance

Excess water kefir (WK) grains were weight out and frozen in 15 ml falcon tubes at -20°C for a minimum of 2 days. Frozen grains were thawed at 4°C for 2 hours and transferred into 4°C cold, fresh WK media. Grain growth was assessed by weight 48h after the start of the fermentation.

Identification of possible AMO and POD coding genes in WK MAGs

Experimentally validated ammonia monooxygenase (AMO; Q04507.2, Q04508.2, and Q82W83.1) and pyruvic oxime dioxygenase (POD; GAU72725.1) sequences were downloaded from NCBI. Predicted AMO protein sequences were downloaded from UniPort.org. Bacterial and fungal MAGs were used to create a blast nucleotide database. The blast database was queried with the AMO and POD sequences using the tblastn function ([1], v. 2.14.1).

Supplementary Results and Discussions

Sequencing results

For each of the 69 WK grains, five samples (2x 8h liquid, 2x 48h liquid, 1x 48h grains) were the subject of shotgun sequencing. An additional nine media controls (functioning as negative controls) and two positive controls (the same sample in different sequencing runs; WK002-2-G-48h) were sequenced. A total of 1.3×10^9 reads and 3.8×10^{11} bp were obtained. Of these, 99.5% of the reads and 94.3% of the bp passed quality control. Media controls did not show a PCR product during sample preparation and yielded an average of 1007 reads per sample, while WK samples yielded an average of 3.7 million reads per sample (Supplementary Data 1).

Development and validation of a water kefir specific InStrain data base

We initially attempted species-level taxonomic profiling using the commonly used taxonomic profilers Kraken 2[2] & Bracken[3], Kaiju[4], and MetaPhlAn 3[5] with their respective default databases, but had limited success in the profiling of the WK metagenomes (data not included), given the high number of novel species and the difficulties of defining true positives and false positive detections in this not well studied environment. Verce et al., highlighted similar difficulties using standard taxonomic profilers for WK and therefore used a metagenomic recruitment plot approach to detect species in the WK microbiome[6]. While their metagenomic recruitment plot approach provides visually convincing evidence for species detection, it is not very scalable. We therefore used inStrain for taxonomic profiling, as it has been shown to be a reliable taxonomic profiler, providing additional genome breadth and genome coverage information, and allowing for a stricter definition of detection[7-9], as compared to a, for example, simple relative abundance based approach for detection[10].

Kaiju was used for an initial taxonomic profiling at genus-level to provide the foundation for the creation of a custom WK inStrain database. Kaiju is a fast DNA-to-protein profiler with a comprehensive database, but is suboptimal at species-level classification[11]. Nevertheless,

Kaiju provides a good overview of the genera present within the samples. Kaiju detected 38 bacterial and 10 fungal genera that were present in at least one sample with >1% relative abundance. Two genera (*Ethanoligenens* and *Pseudoclavibacter*) were detected through MAG assembly even though their relative abundance, as determined by Kaiju, was less than >1%. The representative of *Ethanoligenens* was detected in three different WKs and appeared to be a novel species. The *Pseudoclavibacter* MAG was only detected in one WK and also responds to a putatively novel species. Overall, 19/40 bacterial genera and 6/10 fungal genera identified using Kaiju were also represented by at least one MAG. To facilitate improved taxonomic assignment, a custom database was generated. Firstly, NCBI reference genomes were downloaded for the 40 bacterial and 10 fungal genera. Reference genomes were reduced to species representative genomes to facilitate taxonomic profiling with inStrain. Bacterial reference genomes and bacterial MAGs from this study were de-replicated to species level representative genomes using dRep and fungal reference genomes were compared using FastANI and manually curated. The genome from a *Leuconostoc pseudomesenteroides* isolate and a *Leu. pseudomesenteroides* MAG genome were both retained, as they shared less than 95% ANI with each other. The inStrain species representative database created contained 1787 NCBI reference genomes and 39 bacterial MAGs. A list of the 1826 genomes that are included in the inStrain database is given in Supplementary Data 1.

We have applied a genome breadth cut-off of 0.35, meaning that 35% of the genome needed be covered with at least one read to be considered as present, which is slightly more lenient than the suggest 50% genome breadth cut-off[7], but we have added an additional cut-off based on the expected breadth to observed breadth ratio, requiring a minimum ratio of 0.75. This ratio cut-off allows the removal of genomes with high coverage, but lacking major parts of the genome, e.g., genomes that are expected to have nearly 100% breadth based on the given coverage would be removed if less than 75% of the genome is detected. At the same time, the 0.75 ratio cut-off allows leniency for genomic differences between the species in the WK

and species representative genome in the database, as well as sequencing biases. The 0.75 ratio cut-off was empirically determined for this data set. The removal of potential false positive species, that does not follow the statistical expectations for the expected breadth (breadth = $1-e^{-0.883*coverage}$; [7]), can be seen in Supplementary Figure 16. We are aware that these strict cut-offs might remove low abundant species, but are willing to accept this for increased reliability in the species that we are detecting. The metabolic contribution of very low abundant species to the final beverage might be questionable during the fermentation anyway. The lowest fungal and bacterial species detected with the given cut-offs were *Pi. membranifaciens* and *A. indonesiensis* with 0.109% and 0.136% relative abundance, respectively, which is close to the previously recommended relative abundance thresholds of <0.1% for food metagenomic samples[10].

Water kefir grain growth and microbial associations

WK grains are made up off mostly dextran, primarily made of (1→6)-α-D-glucan[12]. Differently branched dextrans and lower amounts of levans suggest the involvement of multiple species in WK EPS production[13]. Figure 6 showed positive correlations for several species with grain growth, suggesting as well, that several species might be involved in WK grain growth. *Len. hilgardii* has been shown to produce large amounts of dextran and its dextransucrase, responsible for dextran production, has been characterised[14]. *Len. hilgardii* showed here the strongest correlations with grain growth (Figure 6). Dextransucrases from *Li. hordei* and *Li. nagelii* (isolated from WK) have been characterised as well[15] and dextran production has been shown for *Leu. mesenteroides*[16]. *Li. nagelii* and *Leu. mesenteroides* showed strong positive correlations with grain growth as well (Figure 6). Further microbes, in particular LABs, might be involved in dextran production in WK, as the Glycoside Hydrolase Family 70 (GH70; containing dextransucrases), are nearly exclusively present in the order *Lactobacillales* (cazy.org)[17].

Drying of WK grains is a common practice, recommended in various WK community forums, allowing easier shipping and sharing of WK grains. In this study we observed that dried WK

grains had significantly reduced WK grain growth, compared to grains that were sent fresh (Figure 1g). Alpha diversity measures (Shannon & Simpson index) were reduced in WKs derived from dried grains (Figure 4a & b), suggesting a reduced microbial recovery as the cause for reduced grain growth after drying of the grains. In the correlation analysis between species and WK grain growth (Figure 6), the LABs *Len. hilgardii**, Lact. paracasei*, Li. nagelii*, Liquorilactobacillus WK059_bin.2*, Oenococcus WK015_bin.4, Liquorilactobacillus mali A*, Leu. mesenteroides*, Schleiferilactobacillus harbinensis*, Leuconostoc holzapfelii,* and *Lentilactobacillus diolivorans** showed significant positive correlations with grain growth, widening the potential list of microbes involved in dextran production and WK grain growth. Interestingly, *Zym. mobilis* also showed positive correlations with grain growth as well (Figure 6). However, WK042, containing high amounts of *Zym. mobilis* (81.6% relative abundance in the grain sample) and no LABs, did not show any grain growth, suggesting that while *Zym. mobilis* has positive correlations with grain growth, it does not allow grain growth by itself. It was notable that the relative abundance of the LAB species marked with a "*" above were significantly reduced in samples from dried WK grains compared to samples from WK grains that were sent fresh (Supplementary Figure 13). These observations suggest that the common practice of drying grains will negatively impact future grain growth and microbial diversity, which will therefore negatively impact future fermentations.

Water kefir grain growth is not impacted by freezing

Excess grains of well-growing grains were frozen at -20°C to test the freeze tolerance as a possible means of preserving WK grains. Grains that were frozen, for a minimum of two days and up to several months, were not significantly impacted in their grain growth by freezing, defrosting, and starting a new fermentation, as grain growth was not significantly different to before freezing (n = 32; Supplementary Figure 2).

A study with industrially used WK grains observed that freezing and thawing damaged a batch of WK grains and that the grains did not show any signs of grain growth subsequently[18]. We tested the freeze tolerance of 32 WK grains with excess grains and did not observe a

significant impact of freezing on WK grain growth (Supplementary Figure 2). These differences could be due to storage duration or inoculum specific. The combined results suggest that freezing of WK grains should the preferred method over drying of grains for the preservation of communities, even though further analysis of the microbial communities after freezing would be desirable.

Species co-occurrence analysis

Species co-occurrence analysis showed mostly random (1105/1210), some positive (85/1210), and few negative (20/1210) co-occurrences between pairs of species (Supplementary Figure 15). *Bi. aquikefiri* (14 positive, 3 negative), *Zyg. florentina* (10 positive, 1 negative), *Li. nagelii* (8 positive, 2 negative), *A. orientalis* (8 positive, 2 negative), *Liquorilactobacillus WK059_bin.2* (9 positive), *Liquorilactobacillus ghanensis* (9 positive), *Lact. paracasei* (9 positive) are the species with the most significant co-occurrences. *Bi.* WK044_bin.6 was the *Bifidobacterium* with the second most significant co-occurrences, showing 8 positive co-occurrences. *Zym. mobilis* also showed 8 positive co-occurrences while *Sa. cerevisiae* showed 4 positive and 3 negative co-occurrences.

The increase of species co-occurrence, above random chance, can indicate positive interactions (mutualism (+/+) or commensalism (+/0)) between pairs of species, while a decrease in species co-occurrence can indicate negative interactions (competition (-/-) or amensalism (-/0)). The effect of parasitism (+/-) depends on the dependency between species[19,20]. Species co-occurrence analysis suggests that most of the co-occurrences in WK are random. Significant co-occurrences are mostly positive and the fewest co-occurrences are negative. Bifidobacteria, in particular *Bi. aquikefiri* and to a lesser degree *Bi.* WK044_bin.6, seem to play an important role in the WK community, as they are characterized with a high number of positive co-occurrences (Supplementary Figure 15) and are very prevalent in WK (40.58 and 27.54% prevalence, respectively). For example, *Bi. aquikefiri* shows positive co-occurrences with *A. okinawensis, A. orientalis, Bi. WK044_bin.6, Lact. paracasei, Len. hilgardii, Leu. mesenteroides, Li. ghanensis, Li. nagelii, Li. WK059_bin.2, Li. satsumensis, O.*

WK015_bin.4, Zyg. florentina, Zym. mobilis, and *Zym. pomaceae.* While only showing negative co-occurrence with *Glb. sp. Gdi, Lach. fermentati,* and *O. oeni.* Several LAB species also show significant positive interactions. Interestingly, *Zym. mobilis* does not show any negative co-occurrences (Supplementary Figure 15), even though it is highly dominant (Supplementary Figure 4, Supplementary Table 1), shows negative correlations with alpha diversity measures (Figure 6), and *Zym. mobilis* dominated communities shows a distinct pattern in beta diversity analysis (Figure 5b and c). While the co-occurrence analysis does not allow direct insights into the type of interaction (mutualism, commensalism, competition, amensalism, or parasitism), it highlights key species within the microbial network[19] and is a foundation for future targeted investigations of species pairs and their interactions.

The particular role of *Zymomonas mobilis* in water kefir

In this study we have detected *Zym. mobilis* and *Zym. pomaceae* (emended to *Zymomonas mobilis* subsp*. pomaceae*[21]). *Zym. mobilis* has gained scientific interests, as it is an even more efficient ethanol producer than *Sa. cerevisiae*[22]. Interestingly, *Zym. mobilis* has been absent (or nearly absent) in some WK studies[6,23,24] and highly abundant in other studies[25,26]. *Zymomonas* dominated WK communities cluster particularly far away from other WK communities on genus and species level beta diversity analysis (Figure 5b and c), highlighting how different these WK communities are to the other rather LAB or AAB dominated community types. *Zymomonas* has a relatively low occupancy (33.3%), but *Zym. mobilis* and *Zym. pomaceae* are highly abundant when present (Figure 5a, Supplementary Figure 4, Supplementary Table 1). *Zym. mobilis* showed some of the strongest negative correlations with alpha diversity measure (Figure 6), likely due to its high relative abundance. Interestingly, *Zym. mobilis* and *Zym. pomaceae* were nearly absent in samples from dried WK grains (Supplementary Figure 13), suggesting a sensitivity to the conditions during drying. The metabolite analysis shows that the WK fermentations cluster primarily by the fermentation time and the *Zym. mobilis* dominated WK WK042 at 48h sample does not cluster away from the other 48h samples (Supplementary Figure 6). While rather unremarkable in its metabolite

profile (Figure 8), *Zym. mobilis* showed positive correlations with methyl dodecanoate and methyl isobutyl ketone (described as waxy, soapy, creamy, coconut, mushroom and sharp, solvent, green, herbal, fruity, dairy, spicy, respectively) in the volatile organic compound analysis (Figure 10) and the *Zym. mobilis* dominated WK042 48h sample shows a very distinct volatile organic compound profile, clustering away from all other WK samples (Figure 9). These results suggest that *Zym. mobilis* contributes to a distinct WK flavour profile.

Observation of individual cases of contaminations and undesired species

Some of the low prevalent species could be species that are not necessarily desired in WK. For example we have detected three different *Clostridium* species using inStrain. *Clostridium acidisoli,* initially isolated from acidic peat-bog soil[27], *Clostridium diolis* (reclassified to *Clostridium beijerinckii*[28]), isolated from decaying straw and compost[29,30] and a putatively novel species, *Clostridium I* WK069_bin.5. While there are about 180 known *Clostridium* species, only some are pathogenic[31], and there is very little know about the *Clostridium* species detected in this study. Nevertheless, clostridia are generally undesired in foods and considered a contamination[32,33]. Clostridia generally need anaerobic conditions for growth[31]. A decrease in pH to <4.6 is sufficient to inhibit growth of the food pathogen *Clostridium botulinum*[34]. The WK pH of 3.6 should be sufficient to inhibit the growth of many *Clostridium* species therefore. Clostridia have been previously detected in WK, and their growth had been suggested to be due to the shipping of grains and microbial analysis without pre-fermentations[35]. The highest abundance of *Clostridium* was detected in WK069, a WK that was sent from Hong Kong to Ireland and previously had been fermented anaerobically. The long shipping duration (83 days in this particular case) and the previous anaerobic fermentations, which leads to less of a pH reduction[36], could have favoured *Clostridium* growth. The two pre-fermentations and aerobic growth conditions might not have been sufficient for this particular set of grains to reduce *Clostridium* abundance below detection. Overall, *Clostridium* had a low occupancy with 2.9% prevalence. Another example of undesired microbes in WK is *Paenibacillus polymyxa C. Pa. polymyxa C*, generally not

considered a food associated microbe but a soil microbe[37], has only been detected in WK008. According to the provider of WK008, the grains had been dried for 12 months before they were shared with us. This might have weakened the WK community, allowing *Pa. polymyxa C* to grow. The only other species detected in WK008 were *Gluconobacter japonicus* and *Gluconobacter sp. Gdi*. WK008 needed particularly many pre-fermentations before any fermentation characteristics were noticed. All other WK grains were freshly prepared for this study. The detection of these undesired microbes stresses the importance of good fermentation practices, including successful lowering of the pH, for safe WK production.

Detection of pyruvatoxime

Pyruvatoxime (also referred to as pyruvic oxime or pyruvic acid oxime), is a nitrite-related product, which to our knowledge has not been detected in water kefir before and is not commonly reported in foods. Therefore, the further validation of pyruvatoxime in WK with a complementary method would be desirable[38]. More targeted analysis approaches might not have allowed the detected of pyruvatoxime in WK previously.

A PubMed search revealed only investigations by Malečková et al. into the presence of pyruvatoxime in foods. Malečková et al., investigated malts and beers for pyruvatoxime isomers by GC-MS/MS analysis and detected isomer 1 and isomer 2 in the majority of malts and beers[39]. Pyruvatoxime has been found in various human samples such as urine[40], stool[41], and potentially saliva[42].

Pyruvatoxime has been studied primarily in the context of heterotrophic nitrification, a nitrogen cycling process in the environment that is not typically associated with foods. Pyruvatoxime is an intermediary product in the pyruvic oxime dioxygenase (POD) dependent pathway. During heterotrophic nitrification, ammonia is converted to hydroxylamine (NH_2OH) by the ammonia monooxygenase (AMO)[43]. Hydroxylamine reacts than spontaneously with pyruvate to pyruvatoxime. Pyruvatoxime is oxygenated by POD to pyruvate and nitrate[44,45]. AMOs, while mostly studied in ammonia-oxidizing archaea and ammonia-oxidizing bacteria, have been

predicted in fermented food associated bacteria, such as *Komagataeibacter xylinus* (GenBank ID PYD58067.1) and *Komagataeibacter saccharivorans* (GenBank ID AXY24032.1). Both species have been detected in this study as well (Supplementary Table 1). Possible POD coding genes, for the oxygenation of pyruvatoxime, have been found in Proteobacteria, Actinobacteria and Ascomycota[46]. A tblastn search with the experimentally validated AMO (Q04507.2, Q04508.2, and Q82W83.1) and POD (GAU72725.1) sequences, as well as predicted AMO sequences, revealed possible AMO and POD coding genes within the WK MAGs (Supplementary Table 2). AMO coding genes were primarily predicted within AAB and *Zymomonas,* especially based on the previously predicted AMO proteins. Possible POD related proteins were predicted within several yeasts and *A. papayae* (Supplementary Table 2). Tsujino et al. used E-values of <e-60 for POD homolog discovery, according to which no POD homologs would be present within the WK MAGs.

The potential role of pyruvatoxime in nitrogen cycling within WK warrants further investigation and the use of a complementary detection method is desirable. Given that WK fermentations are low in nitrogen[47,48], potentially limiting microbial growth, this presents an intriguing system for further exploration.

Supplementary Figures

Supplementary Figure 1. Kefir grain growth depends on inoculums.

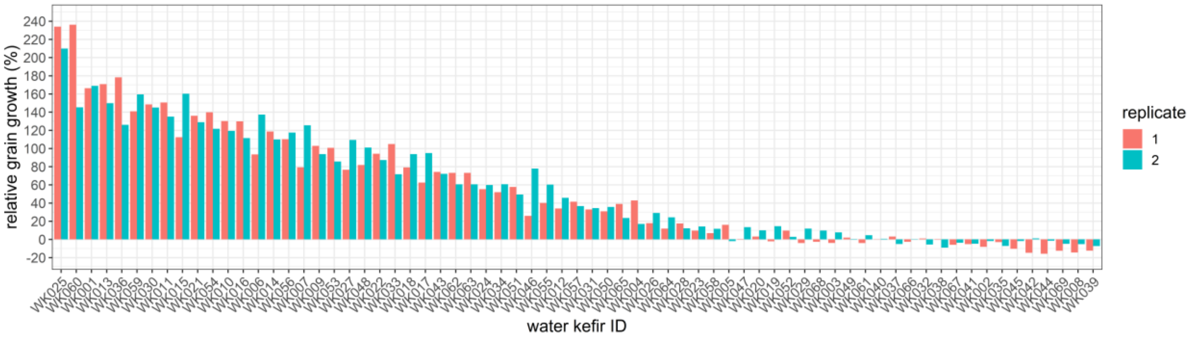

Relative grain growth was determined based on grain weight before and after 48h of fermentation for the two experimental replicates.

Supplementary Figure 2. Kefir grain growth is not significantly impacted by freezing.

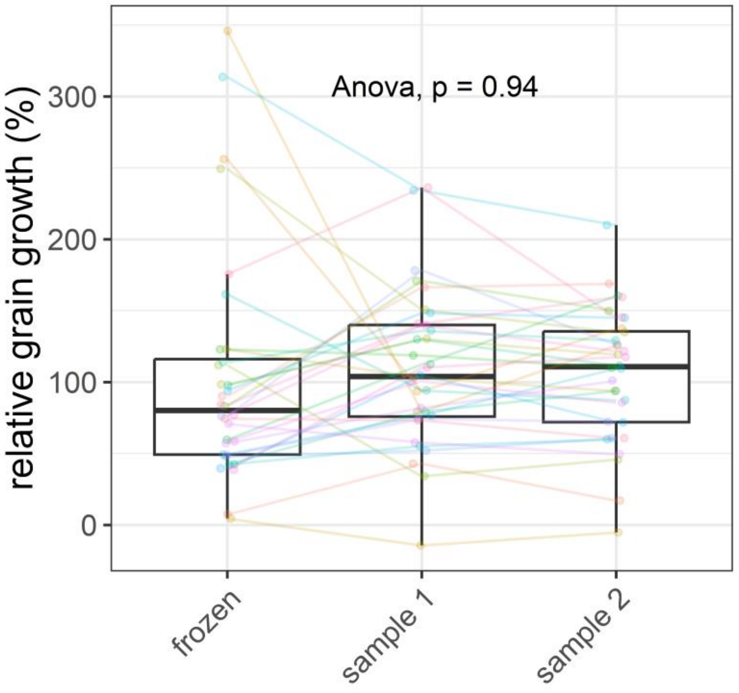

Excess kefir grains (*frozen*) from *sample 1* were frozen for a minimum of two days to test for freeze tolerance and used for a new fermentation after defrosting. *Sample 1* and *sample 2* indicate the two experimental replicates of the grain set that were not frozen. A total of n = 32 grain sets were tested.

Supplementary Figure 3. Number of species per sample.

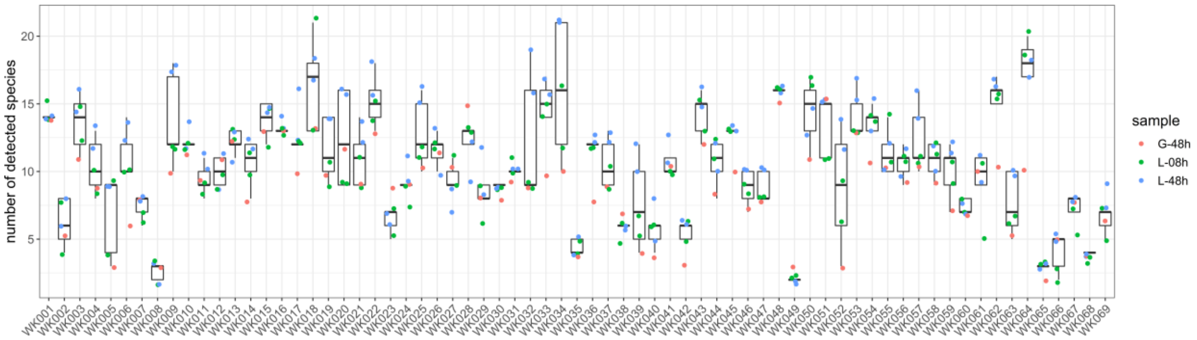

InStrain was used for the detection of species. The average is 10.3 species per sample, with a trend towards a higher number of detectable species in the 48hr liquid samples. G-48h, grain samples taken after 48h of fermentation; L-08h, liquid samples taken after 8h of fermentation; L-48h, liquid samples taken after 48h of fermentation.

Supplementary Figure 4. Relative abundance of species within WK

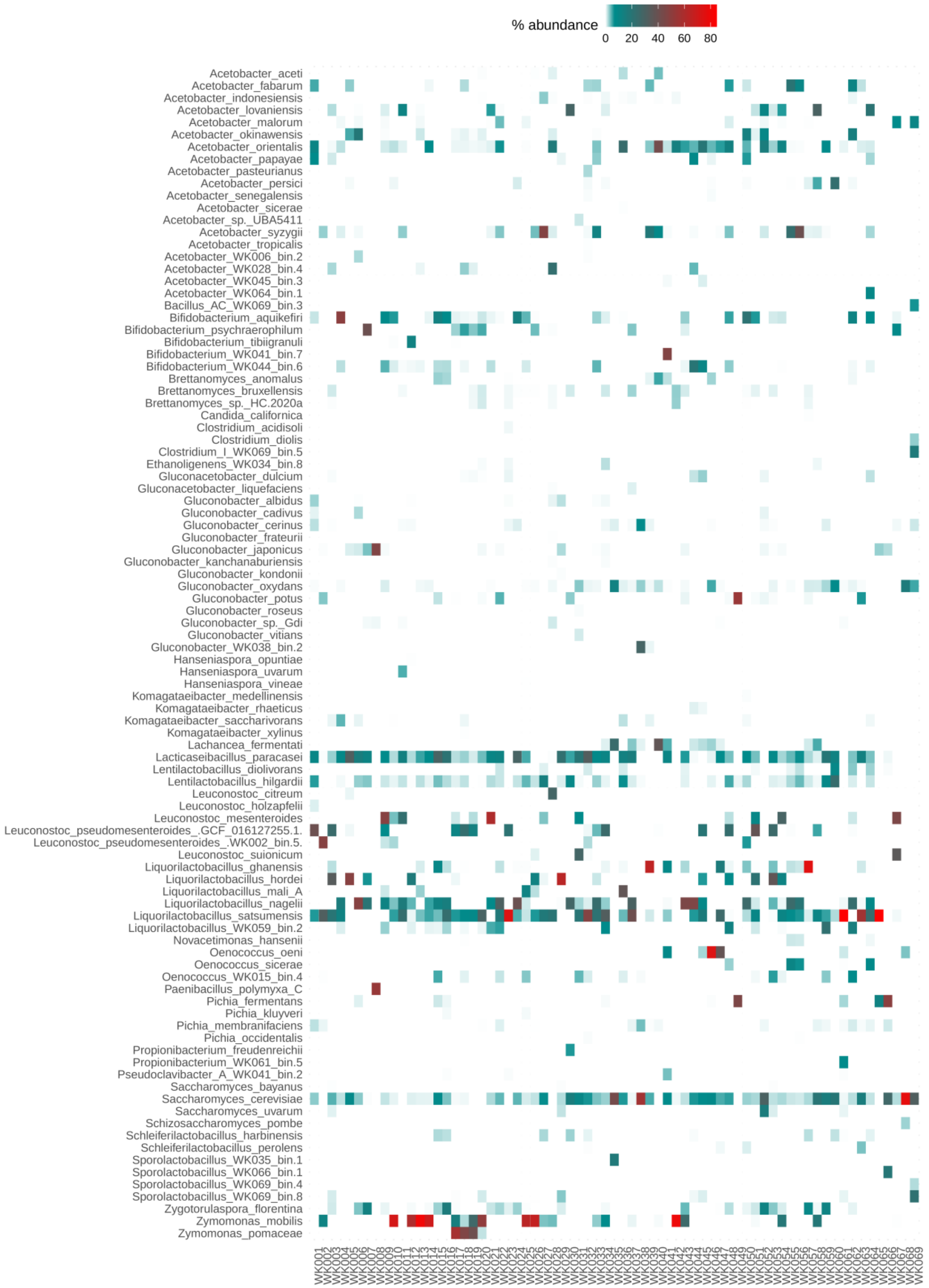

Heatmap showing the relative abundance of species in the different WK. Relative abundance is given as the average of the five samples (grains, 2x liquid 8h, 2x liquid 48h) for each of the 69 WKs.

Supplementary Figure 5. Impact of fermentation practices (self-reported) on alpha diversity.

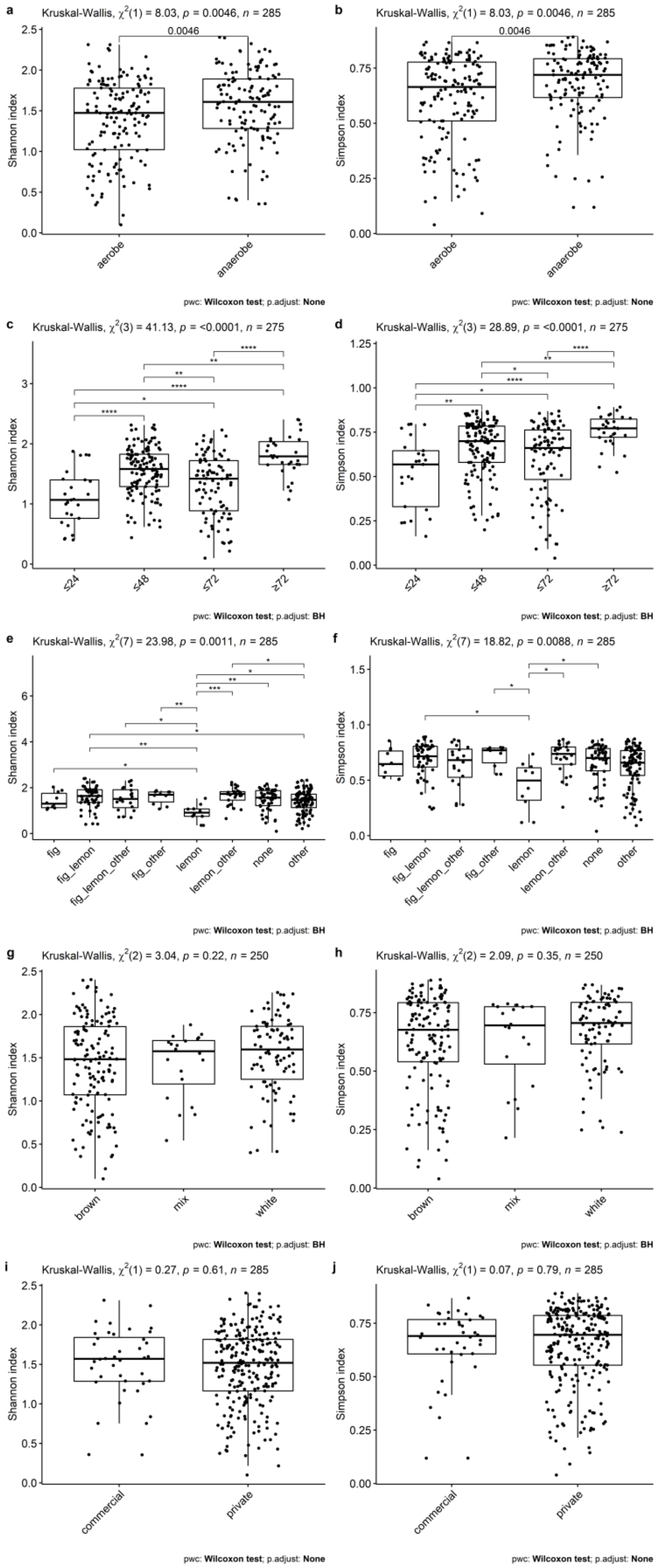

WK grain providers self-reported previous fermentation practices. Previous fermentation practices and their impact on alpha diversity measures (Shannon and Simpson index) was analysed. Aerobic or anaerobic fermentations (a & b); fermentation duration in hours (c & d); additional nutrient source during the fermentation (e & f); sugar type (g & h); commercial or private use of the grains (I & j).

Supplementary Figure 6. Heat map of detected metabolites.

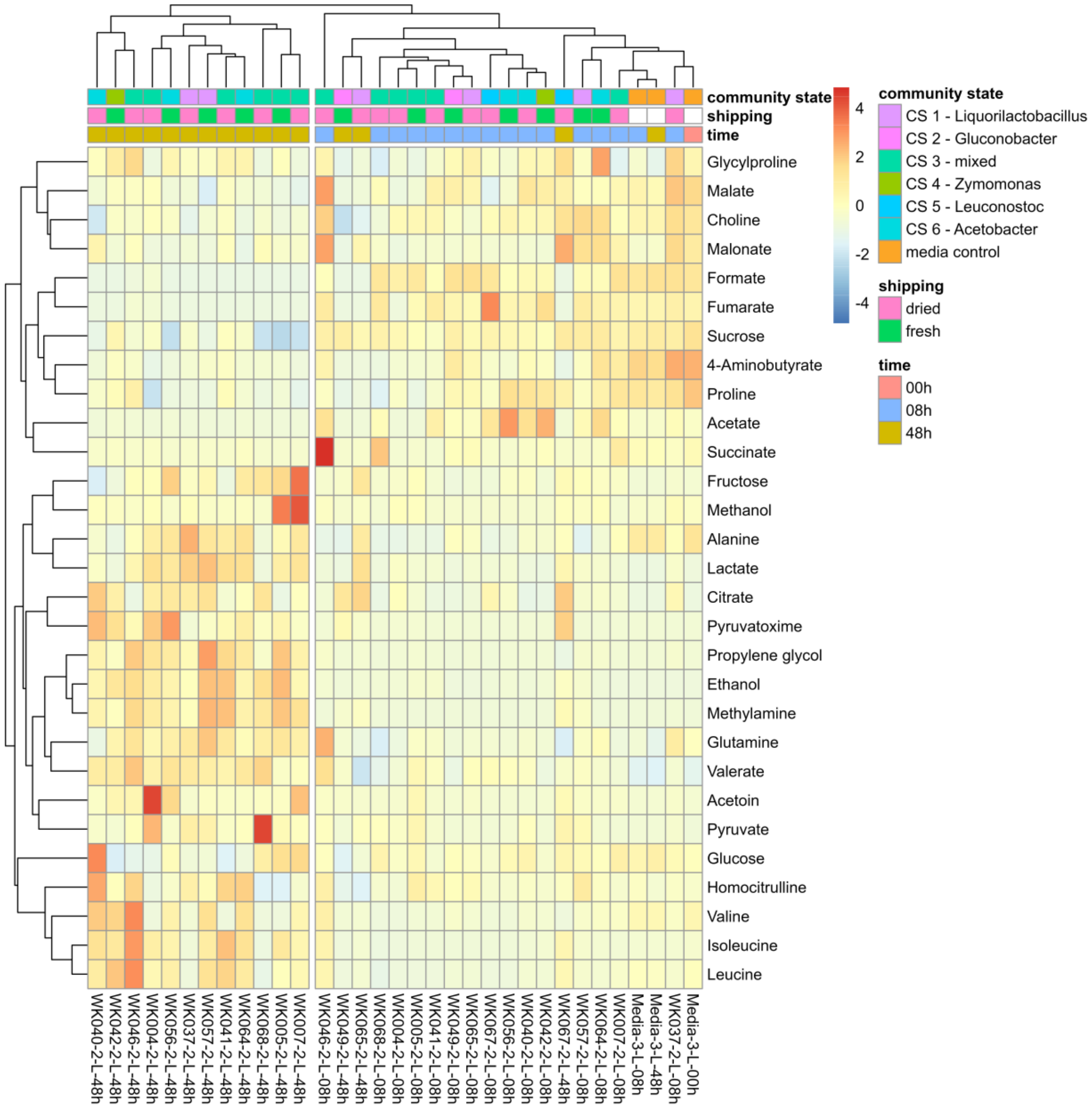

Heat map of metabolites that were detected by NMR analysis, scaled by metabolite. Samples cluster primarily by the sampling time point (8h or 48h), with less of a discernible clustering by the community state or how the grains were shipped (dried or fresh).

Supplementary Figure 7. Heat map detected metabolites. Boxplot of metabolites showing statistical differences between metabolite abundances during the sampled time points of the fermentatio

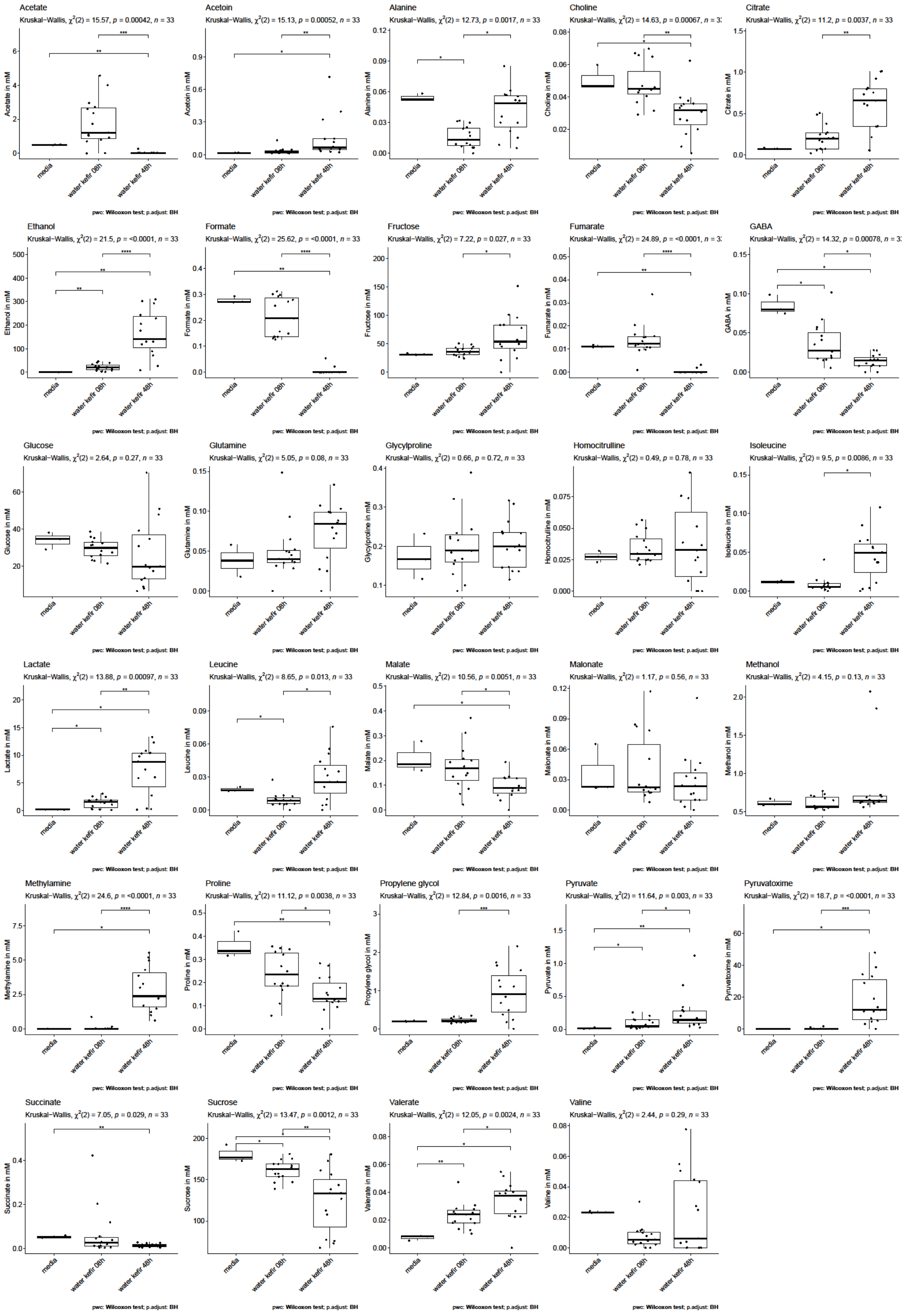

Media n = 3; WK 08h n = 15, WK 48h n = 15.

Supplementary Figure 8. Stacked bar plots of metabolies shows the accumulatinon of alcohols and organic acids during the sampled time points of the fermentation.

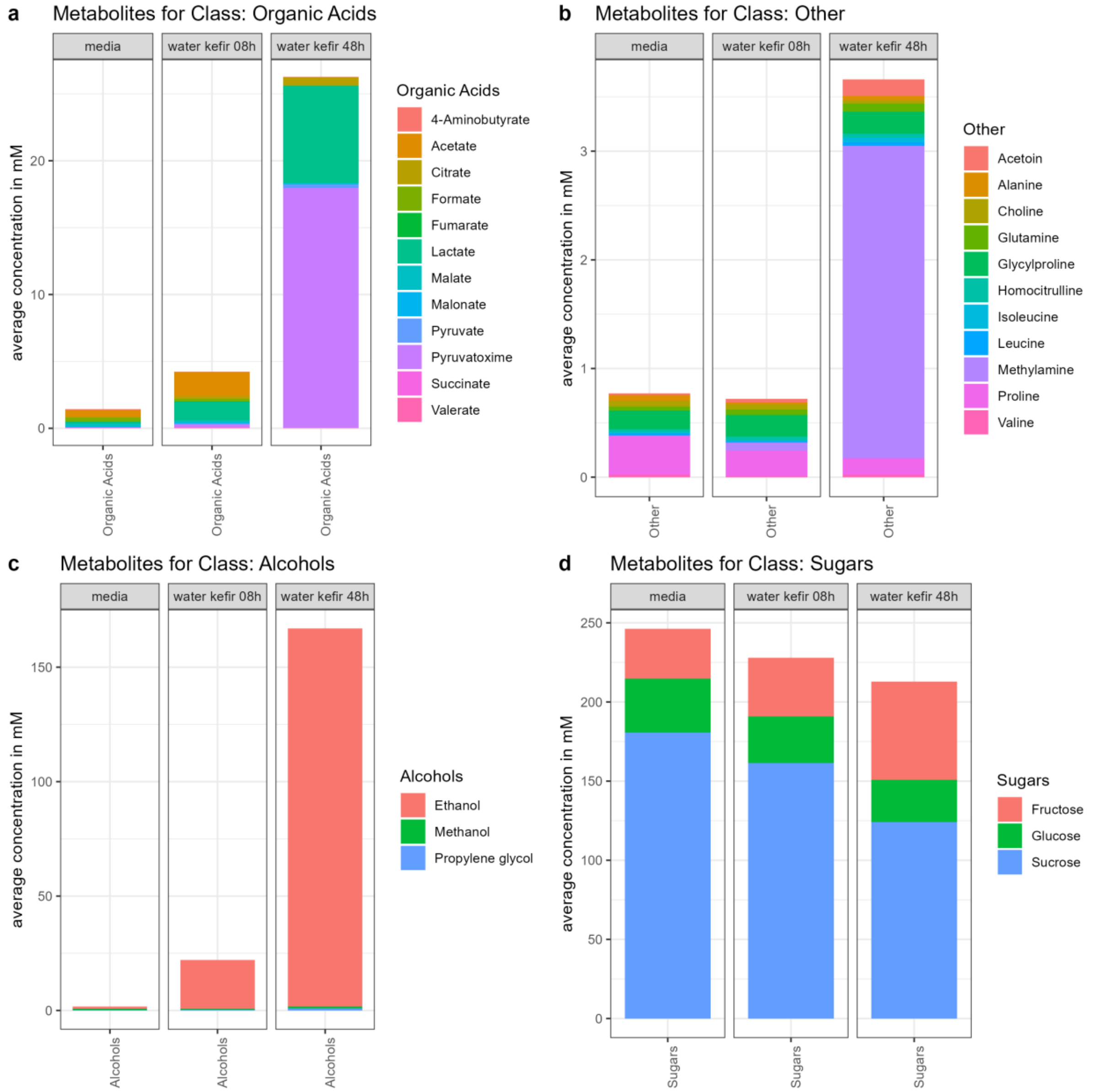

Stacked bar plots of metabolites detected by NMR analysis, grouped by organic acids (**a**), other (**b**), alcohols (**c**) and sugars (**d**). Media is the average of three samples, while the WK 08h and 48h samples are the average of 15 samples each.

Supplementary Figure 9. PCoA plot of VOCs using the Canberra distance shows a clear separation between media control, 8h, and 48h water kefir fermentations.

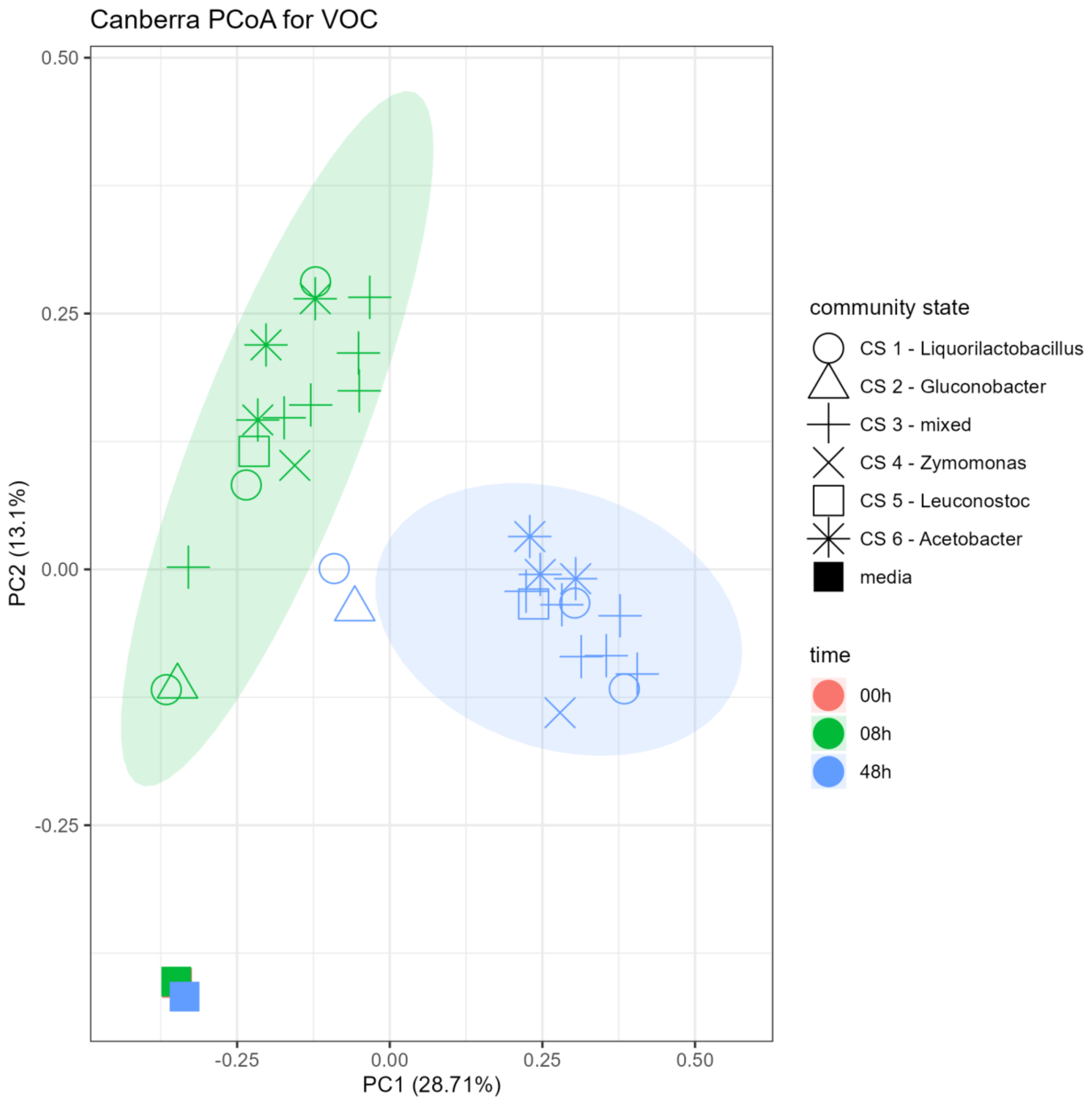

Media n = 3; WK 08h n = 15, WK 48h n = 15. Canberra distance was chosen as it is common for VOC data[49,50].

Supplementary Figure 10. Stacked bar plots of volatile organic compounds shows the increase of aroma compounds during the sampled time points of the fermentation, in particular volatile acids, alcohols, and esters.

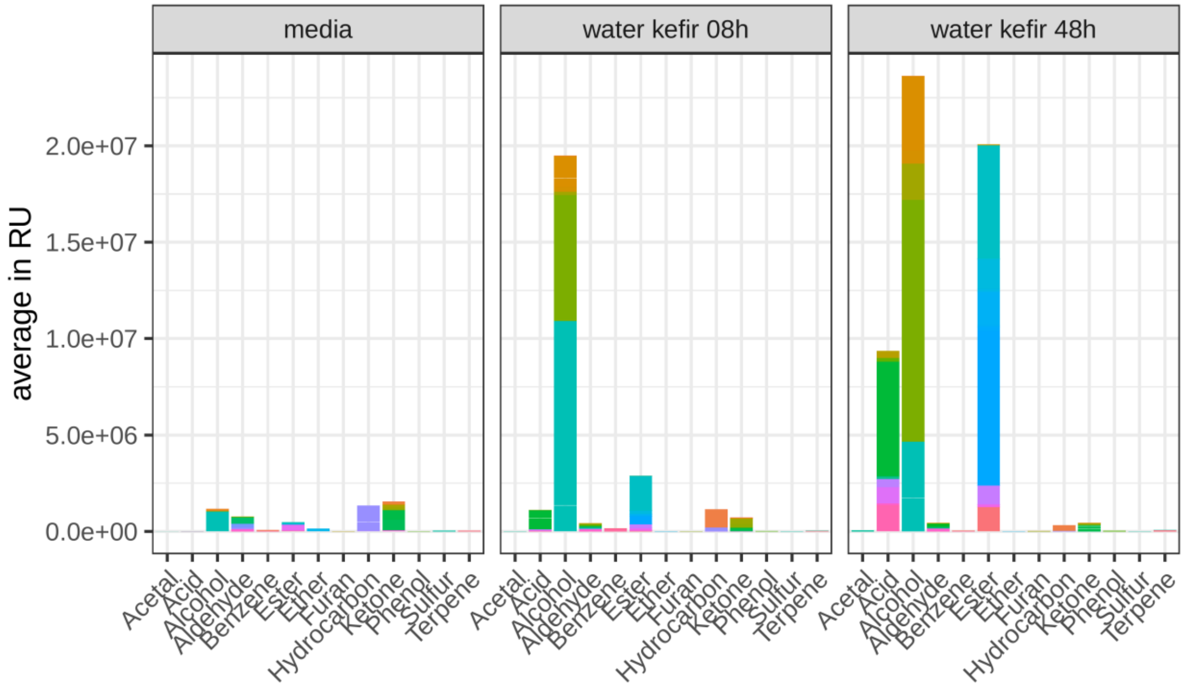

Stacked bar plots of volatile organic compounds detected by GS-MS analysis. Media is the average of three samples, while the WK 08h and 48h samples are the average of 15 samples each. Colours represent different compounds (see Supplementary Data 1).

Supplementary Figure 11. Boxplot of volatile organic compounds detected by GC-MS showing statistical differences between metabolite abundances during the sampled time points of the fermentation.

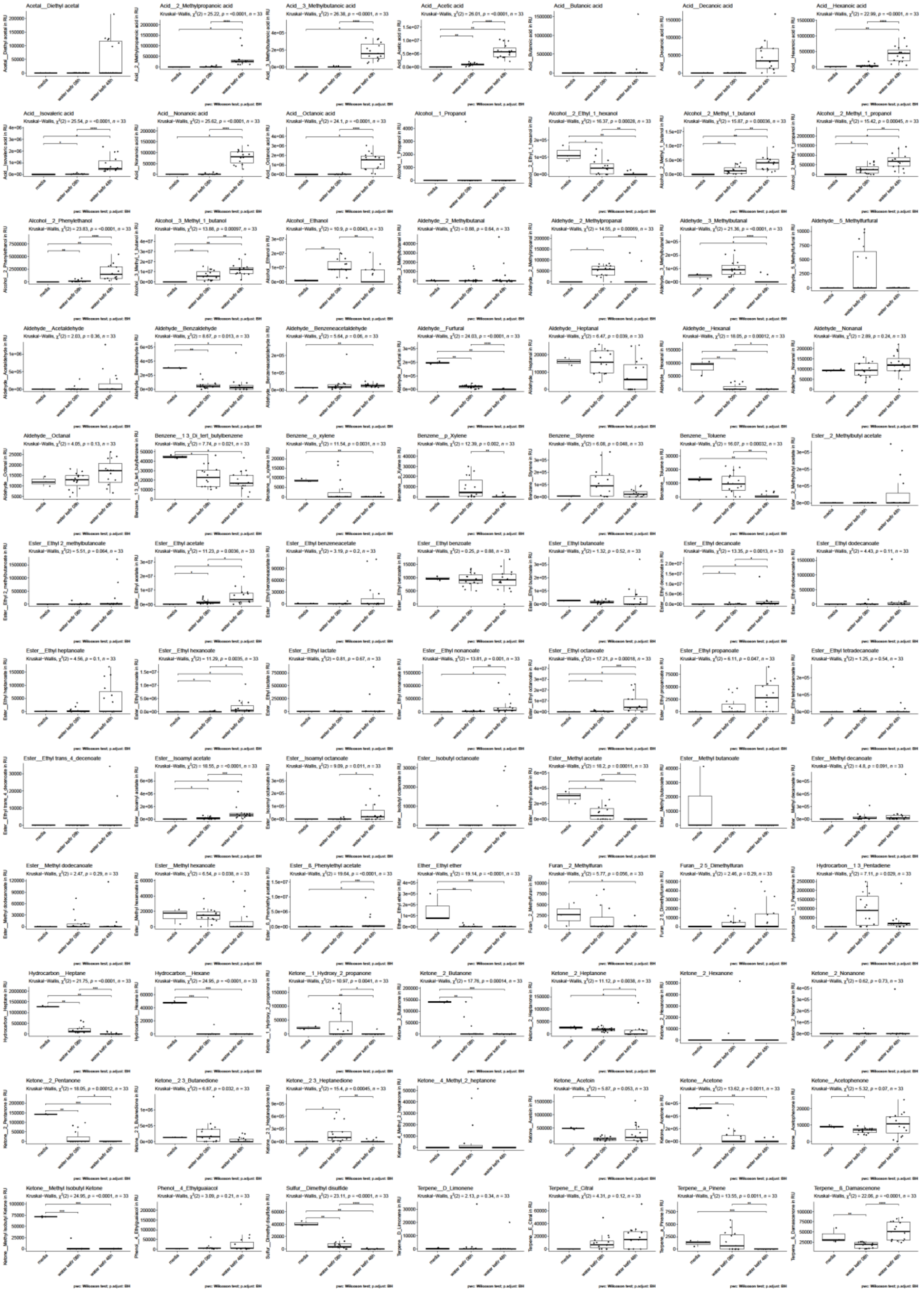

VOC values are given as relative units (RU). The chemical class is followed by the chemical name. Statistical analysis was only performed when at least two groups were detected (e.g. 08h and 48h samples). Media n = 3; WK 08h n = 15, WK 48h n = 15.

Supplementary Figure 12. Multi-factor analysis indicates key drivers for differences between samples.

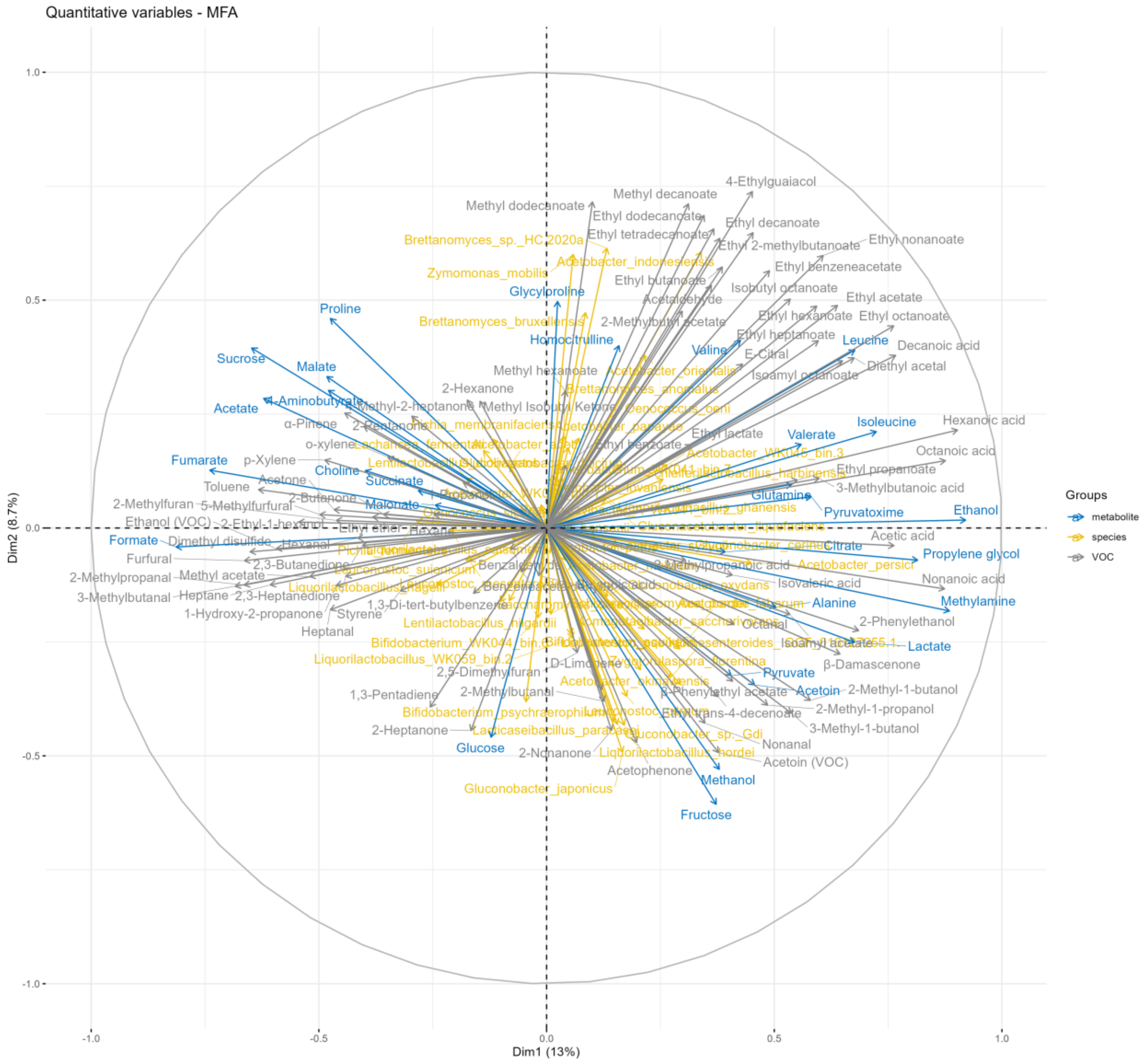

Multi-factor analysis (MFA) of species, metabolite, and volatile organic compound (VOC) abundances.

Supplementary Figure 13. The impact of drying water kefir grains on species abundance.

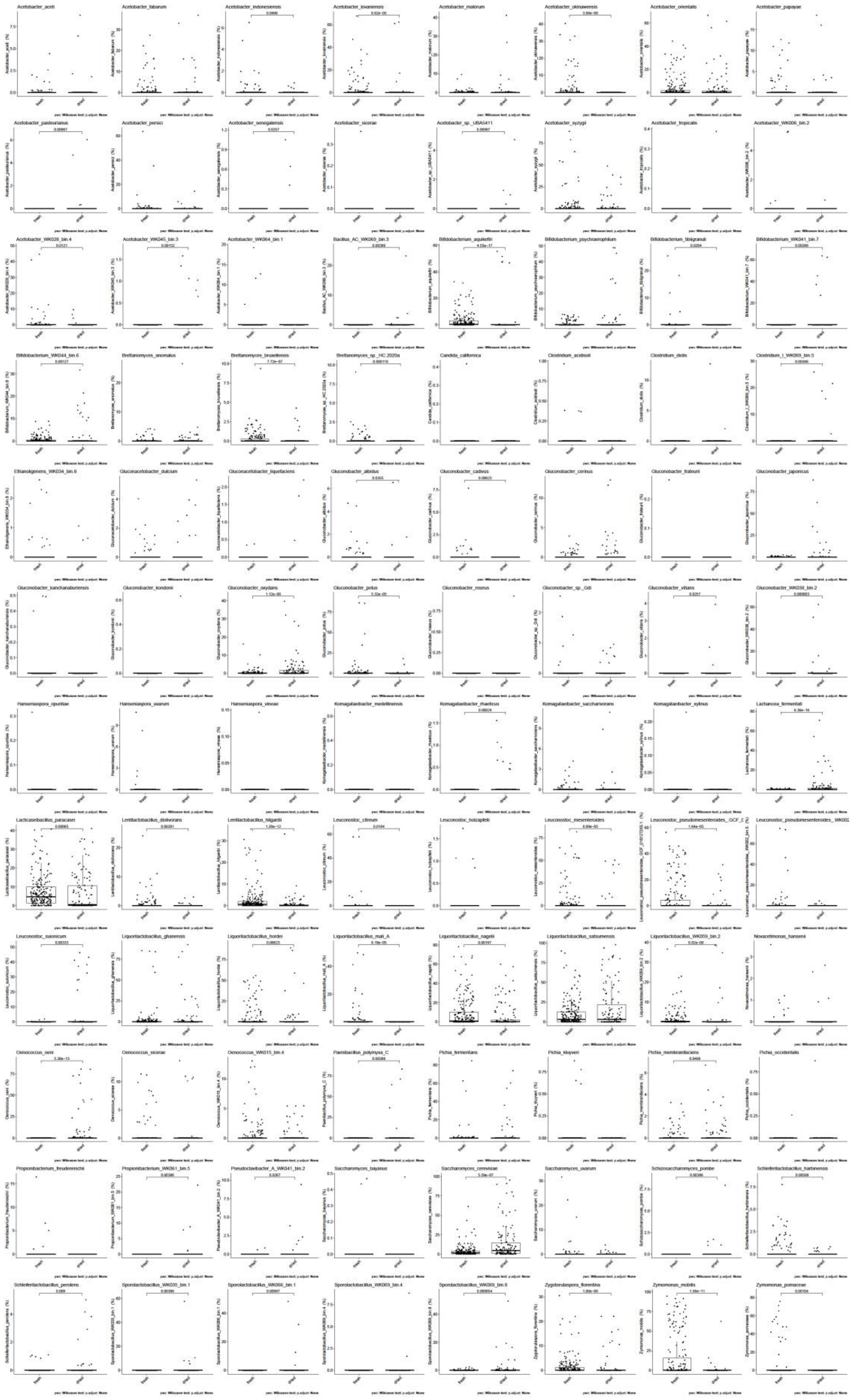

Relative species abundance by samples that were sent fresh or dried to us, show how species are differently impacted by drying.

Supplementary Figure 14. The impact of drying water kefir grains on genus abundance.

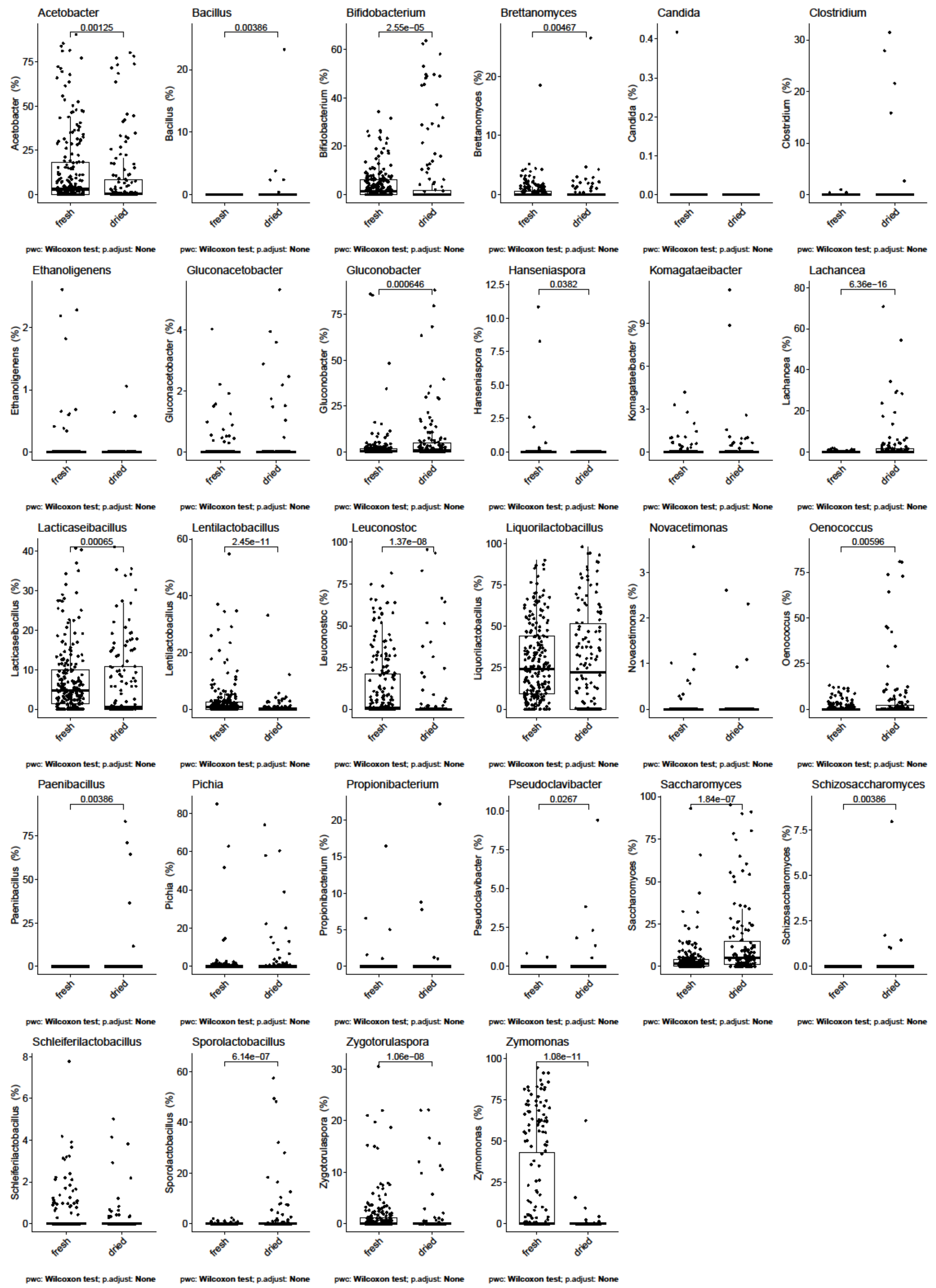

Relative genus abundance by samples that were sent fresh or dried to us, show how genera are differently impacted by drying.

Supplementary Figure 15. Species co-occurrences in water kefir.

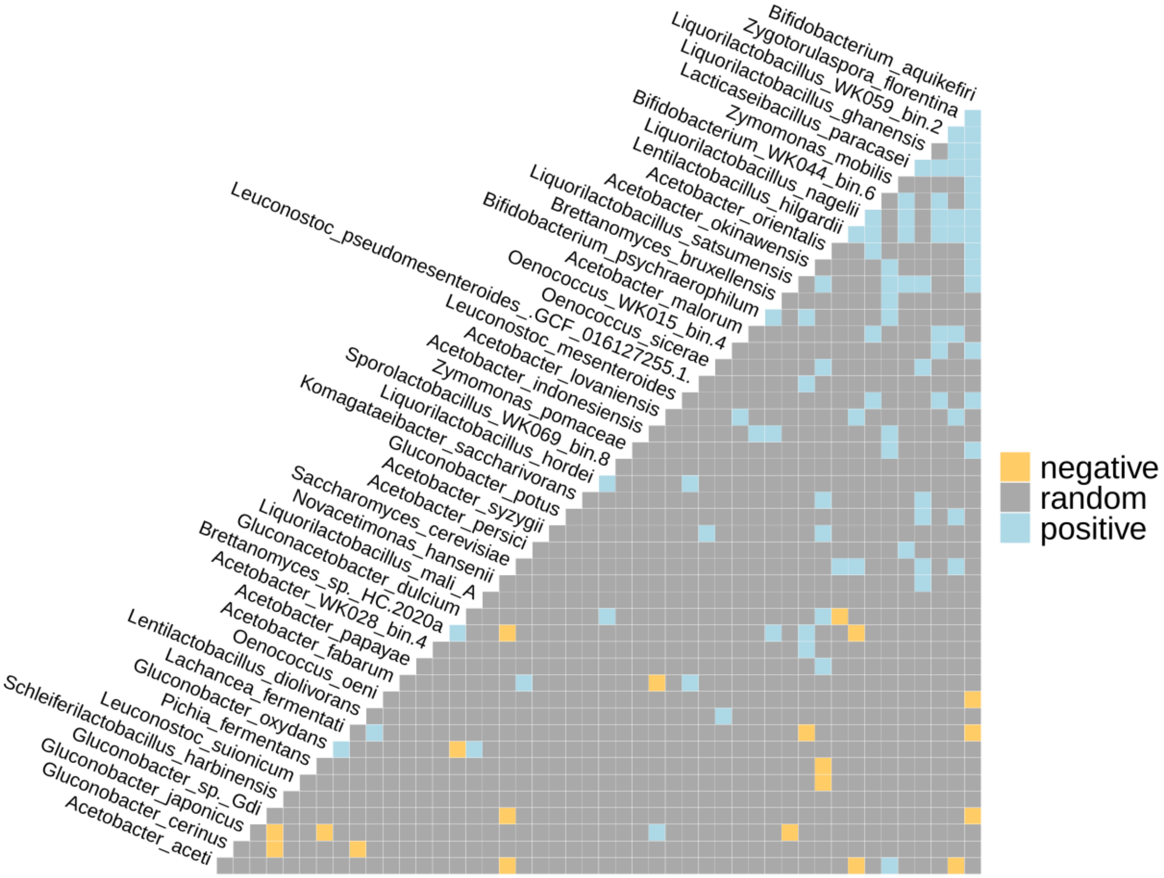

Most co-occurrences between pairs of species are random, while positive co-occurrences are more common than negative co-occurrences.

Supplementary Figure 16. inStrain detected species without and with applying filters.

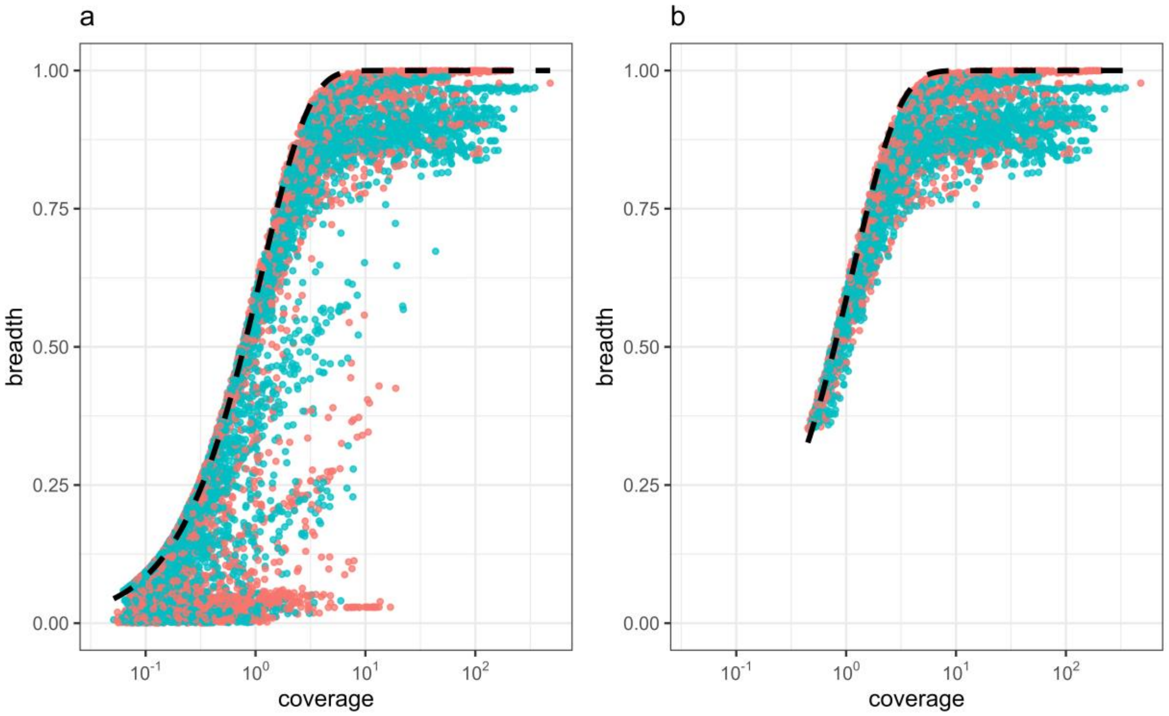

Genome breadth and coverage are given for each species in all samples, with no filters (a), and after applying a filter requiring at least 35% breadth, as well as a minimum ratio of 0.75 for the expected breadth to observed breadth for detection (b). Matches to genomes derived from MAGs are indicated in red and NCBI reference genomes in blue. The dashed line is based on the empirically determined function, describing the expected breadth to coverage ratio[7].

Supplementary Tables

Supplementary Table 1. Prevalence and maximum abundance of species.

group	species	MAG with highest completeness in %	prevalence in %	max. abundance in %	WK number and sample type with max. abundance	previously isolated from water kefir	previously detected in water kefir
AAB	*Acetobacter aceti*	99.4	8.7	8.8	WK040 liquid 48h		[24,35]
AAB	*Acetobacter fabarum*	100	18.84	36.82	WK055 liquid 48h	[36]	
AAB	*Acetobacter indonesiensis*	100	15.94	6.5	WK027 liquid 48h	[36]	[25]
AAB	*Acetobacter lovaniensis*	100	21.74	67.66	WK030 liquid 48h	[51]	
AAB	*Acetobacter malorum*	100	18.84	41.23	WK069 liquid 48h		[24]
AAB	*Acetobacter okinawensis*	100	20.29	40.39	WK006 liquid 48h	[18]	[24]
AAB	*Acetobacter orientalis*	100	53.62	66.86	WK040 liquid 48h	[18,52]	[24]
AAB	*Acetobacter papayae*	99.5	11.59	18.42	WK044 liquid 48h		[24]
AAB	*Acetobacter pasteurianus*	99.5	1.45	6.03	WK032 liquid 48h	[53]	[24]
AAB	*Acetobacter persici*	100	17.39	63.72	WK060 liquid 48h		[24]
AAB	*Acetobacter senegalensis*	ND	2.9	1.05	WK046 liquid 48h		[24]
AAB	*Acetobacter sicerae*	ND	1.45	0.36	WK036 liquid 48h		
AAB	*Acetobacter sp. UBA5411*	ND	1.45	4.71	WK031 liquid 48h		
AAB	*Acetobacter syzygii*	100	34.78	88.45	WK027 liquid 48h		[24]
AAB	*Acetobacter tropicalis*	ND	1.45	0.39	WK032 liquid 48h	[53]	[24,52]
AAB	*Acetobacter WK006_bin.2*	99.37	2.9	5.33	WK006 liquid 48h		
AAB	*Acetobacter WK028_bin.4*	100	8.7	44.62	WK028 liquid 08h		
AAB	*Acetobacter WK045_bin.3*	99.17	2.9	1.58	WK045 liquid 08h		
AAB	*Acetobacter WK064_bin.1*	90.01	1.45	19.23	WK064 liquid 48h		
other	*Bacillus AC WK069_bin.3*	92.51	1.45	23.21	WK069 liquid 08h		
Bifidobacterium	*Bifidobacterium aquikefiri*	100	40.58	55.56	WK004 grains	[54]	[18,23,25,36,55,56]
Bifidobacterium	*Bifidobacterium psychraerophilum*	98.86	15.94	49	WK007 grains		[24]
Bifidobacterium	*Bifidobacterium tibiigranuli*	99.21	4.35	25.51	WK012 liquid 48h	[57]	
Bifidobacterium	*Bifidobacterium WK041_bin.7*	98.78	1.45	63.55	WK041 liquid 48h		
Bifidobacterium	*Bifidobacterium WK044_bin.6*	98.32	27.54	31.75	WK044 grains		
yeast	*Brettanomyces anomalus*	88.2	11.59	26.49	WK040 grains		
yeast	*Brettanomyces bruxellensis*	83.6	26.09	9.37	WK042 grains		[24,25]
yeast	*Brettanomyces sp. HC.2020a*	ND	11.59	9.07	WK042 grains		

yeast	*Candida californica* (renamed to *Pichia californica*[58])	ND	1.45	0.42	WK057 liquid 48h	[59]	
other	*Clostridium acidisoli*	ND	1.45	0.97	WK023 grains		
other	*Clostridium diolis* (reclassified to *Clostridium beijerinckii*[28])	99.19	1.45	12.72	WK069 liquid 48h		
other	*Clostridium I* WK069_bin.5	98.16	1.45	25.91	WK069 grains		
other	*Ethanoligenens* WK034_bin.8	97.32	5.8	2.61	WK034 liquid 08h		
AAB	*Gluconacetobacter dulcium*	99.91	13.04	5.32	WK045 liquid 48h		
AAB	*Gluconacetobacter liquefaciens*	96.02	2.9	2.2	WK037 liquid 48h	[60]	
AAB	*Gluconobacter albidus*	91.54	11.59	6.55	WK029 liquid 48h	[61]	[24]
AAB	*Gluconobacter cadivus*	98.27	4.35	7.67	WK006 liquid 48h		
AAB	*Gluconobacter cerinus*	99.5	24.64	13.04	WK038 liquid 48h	[18,62]	[24]
AAB	*Gluconobacter frateurii*	ND	1.45	0.27	WK028 liquid 08h		[24]
AAB	*Gluconobacter japonicus*	100	24.64	88.3	WK008 liquid 48h		[24]
AAB	*Gluconobacter kanchanaburiensis*	ND	2.9	0.5	WK034 liquid 48h		
AAB	*Gluconobacter kondonii*	ND	1.45	0.63	WK029 liquid 48h		
AAB	*Gluconobacter oxydans*	100	47.83	39.72	WK068 liquid 48h	[62]	[24,63]
AAB	*Gluconobacter potus*	(99.5)*	20.29	86.17	WK049 liquid 48h		
AAB	*Gluconobacter roseus*	ND	1.45	0.93	WK031 liquid 08h		[24]
AAB	*Gluconobacter sp. Gdi*	97.29	8.7	2.54	WK028 liquid 08h		
AAB	*Gluconobacter vitians*	ND	1.45	3.94	WK031 liquid 48h		
AAB	*Gluconobacter* WK038_bin.2	98.38	2.9	62.51	WK038 liquid 48h		
yeast	*Hanseniaspora opuntiae*	ND	1.45	0.32	WK012 liquid 08h		[24]
yeast	*Hanseniaspora uvarum*	ND	1.45	10.85	WK011 liquid 08h		[24]
yeast	*Hanseniaspora vineae*	ND	1.45	0.15	WK025 liquid 48h		[26]
AAB	*Komagataeibacter medellinensis*	ND	1.45	0.63	WK050 liquid 08h		[24]
AAB	*Komagataeibacter rhaeticus*	95.01	2.9	1.56	WK044 liquid 08h		[24]
AAB	*Komagataeibacter saccharivorans*	100	13.04	11.38	WK004 liquid 48h	[62]	[52]
AAB	*Komagataeibacter xylinus*	ND	1.45	0.23	WK018 liquid 08h		[24]
yeast	*Lachancea fermentati*	98.4	17.39	70.82	WK040 grains	[64]	[26]
LAB	*Lacticaseibacillus paracasei*	99.46	75.36	41.05	WK032 liquid 08h	[52,65]	[25]
LAB	*Lentilactobacillus diolivorans*	96.4	18.84	24.13	WK060 grains	[65,66]	
LAB	*Lentilactobacillus hilgardii*	99.38	65.22	35.4	WK027 grains	[16,23,36,55,56,65,66]	[6,25,35,63,67]

LAB	*Leuconostoc citreum*	99.78	2.9	57.81	WK028 liquid 48h	[64]	
LAB	*Leuconostoc holzapfelii*	95.15	1.45	1.73	WK001 liquid 08h		[35]
LAB	*Leuconostoc mesenteroides*	100	18.84	81.63	WK021 liquid 08h	[16,18,36,64,68]	[18,24,67]
LAB	*Leuconostoc pseudomesenteroides* (GCF_016127255.1)	100	27.54	56.46	WK001 liquid 08h	[56]	[25]
LAB	*Leuconostoc pseudomesenteroides* (WK002_bin.5)	100	7.25	74.85	WK002 liquid 08h		
LAB	*Leuconostoc suionicum*	100	5.8	46.33	WK067 liquid 08h		[25]
LAB	*Liquorilactobacillus ghanensis*	99.48	33.33	92.35	WK039 liquid 08h	[59]	[24,35]
LAB	*Liquorilactobacillus hordei*	99.48	21.74	89.09	WK029 liquid 08h	[68-70]	[24,25]
LAB	*Liquorilactobacillus mali A*	99.48	7.25	49.75	WK036 liquid 08h	[66,68]	[24,25,55]
LAB	*Liquorilactobacillus nagelii*	99.48	50.72	78.13	WK043 liquid 08h	[18,23,36,55,56,64,66]	[6,18,24,25,35,55]
LAB	*Liquorilactobacillus satsumensis*	99.48	71.01	97.98	WK065 grains	[18,23,66]	[25,63]
LAB	*Liquorilactobacillus* WK059_bin.2	98.95	23.19	38.61	WK059 liquid 08h		
AAB	*Novacetimonas hansenii* (previously *Komagataeibacter hansenii*[71])	97.83	5.8	3.56	WK056 liquid 48h		[24,25]
LAB	*Oenococcus oeni*	98.13	8.7	80.7	WK046 grains	[66]	[24]
LAB	*Oenococcus sicerae*	98.66	7.25	13.74	WK055 grains		[72]
LAB	*Oenococcus* WK015_bin.4	97.86	20.29	13.08	WK062 liquid 48h		
other	*Paenibacillus polymyxa C*	99.85	1.45	83.19	WK008 grains		
yeast	*Pichia fermentans*	78.4	13.04	84.74	WK049 liquid 08h		
yeast	*Pichia kluyveri*	ND	2.9	0.87	WK025 liquid 48h		
yeast	*Pichia membranifaciens*	83.3	24.64	6.69	WK038 liquid 48h	[18,52]	[24]
yeast	*Pichia occidentalis*	ND	2.9	0.88	WK032 liquid 48h		
other	*Propionibacterium freudenreichii*	98.79	1.45	16.44	WK030 grains		
other	*Propionibacterium* WK061_bin.5	97.48	1.45	22.19	WK061 grains		
other	*Pseudoclavibacter A* WK041_bin.2	92.77	5.8	9.42	WK041 grains		
yeast	*Saccharomyces bayanus*	ND	4.35	0.48	WK029 liquid 48h		[24,25]
yeast	*Saccharomyces cerevisiae*	97.4	85.51	95.26	WK068 liquid 08h	[18,23,36,53,55,56,64,68,73,74]	[6,24-26,55,67]
yeast	*Saccharomyces uvarum*	87.5	7.25	31.6	WK052 grains		[24]
yeast	*Schizosaccharomyces pombe*	ND	1.45	7.95	WK068 grains		
LAB	*Schleiferilactobacillus harbinensis*	98.69	14.49	7.78	WK027 grains	[23,36,55,56,65]	[6,23,55]
LAB	*Schleiferilactobacillus perolens*	99.48	5.8	5.02	WK063 liquid 08h	[66]	

LAB	*Sporolactobacillus* WK035_bin.1	98.45	1.45	57.45	WK035 grains		
LAB	*Sporolactobacillus* WK066_bin.1	95.35	1.45	48.24	WK066 grains		
LAB	*Sporolactobacillus* WK069_bin.4	98.45	1.45	8.82	WK069 liquid 48h		
LAB	*Sporolactobacillus* WK069_bin.8**	98.45	13.04	49.5	WK069 grains		
yeast	*Zygotorulaspora florentina*	97.4	40.58	30.48	WK016 liquid 48h	[18,23,64]	
other	*Zymomonas mobilis*	100	33.33	94.47	WK042 liquid 08h	[75]	[24,25,67]
other	*Zymomonas pomaceae* (emended to *Zymomonas mobilis* subsp. *pomaceae*[21])	100	5.8	75.82	WK017 grains		

*MAG from WK049 was classified as *Gluconobacter oxydans B* (GTDB-Tk: 96.99% ANI) and *Gluconobacter sp. R75629* (NCBI DB: 97.17% ANI). No separate *Gluconobacter potus* MAG was detected.

**Since the initial analysis, a genome with a shared ANI of 98.16% has been deposited with NCBI (GCF_024385605.1), the species itself is still unpublished.

Supplementary Table 2. Identification of possible AMO and POD coding sequences within the WK MAGs

Protein	Query	Hit (coting)	Hit (species)	% identity	Length	Mismatch	Gap open	E-value	Bit score
**AMO** (evidence at protein level)	Q04507.2	No hits							
	Q04508.2	No hits							
	Q82W83.1	Bac_WK032_bin.9.fa_NODE_9_length_127197_cov_15.284422	*Acetobacter pasteurianus*	25.969	258	147	9	2.01E-12	71.6
	Q82W83.1	Bac_WK013_bin.7.fa_NODE_1_length_1357253_cov_1050.808237	*Zymomonas mobilis*	29.258	229	118	11	3.61E-12	70.9
	Q82W83.1	Bac_WK013_bin.1.fa_NODE_1311_length_7967_cov_6.328868	*Acetobacter malorum*	27.381	252	134	10	8.79E-12	69.7
	Q82W83.1	Bac_WK031_bin.4.fa_NODE_123_length_52119_cov_8.789970	*Acetobacter sp002409645*	29.614	233	116	10	3.85E-11	67.8
	Q82W83.1	Bac_WK044_bin.4.fa_NODE_631_length_20665_cov_59.847113	*Acetobacter papayae*	24.809	262	147	9	4.94E-11	67.4
	Q82W83.1	Bac_WK040_bin.1.fa_NODE_211_length_31699_cov_20.794147	*Acetobacter aceti B*	29.487	234	117	10	6.06E-11	67.4
**AMO** (predicted)	PYD77552.1	Bac_WK036_bin.3.fa_NODE_1191_length_8998_cov_17.099743	*Acetobacter aceti B*	96.409	362	13	0	6.04E-176	547
	PYD77552.1	Bac_WK006_bin.8.fa_NODE_1082_length_10073_cov_8.962767	*Gluconobacter japonicus*	96.409	362	13	0	6.51E-176	547
	AJA51213.1	Bac_WK069_bin.5.fa_NODE_594_length_27044_cov_112.655489	*Clostridium I* WK069_bin.5	81.303	353	66	0	1.35E-168	526
	PYD77552.1	Bac_WK044_bin.5.fa_NODE_800_length_16332_cov_10.452110	*Komagataeibacter rhaeticus*	92.655	354	23	1	1.39E-161	506
	KLV28092.1	Bac_WK069_bin.3.fa_NODE_754_length_20315_cov_20.388944	*Bacillus AC* WK069_bin.3	57.057	333	142	1	4.76E-112	363
	PNE51755.1	Bac_WK046_bin.4.fa_NODE_1961_length_6435_cov_5.554702	*Lacticaseibacillus paracasei*	57.509	273	115	1	2.31E-105	340
	ARU96113.1	Bac_WK020_bin.8.fa_NODE_2864_length_3925_cov_7.871318	*Zymomonas pomaceae*	53.731	335	154	1	6.54E-100	326
	PCK80124.1	Bac_WK033_bin.10.fa_NODE_1973_length_4419_cov_26.653758	*Zymomonas mobilis*	46.839	348	176	4	1.48E-89	298
**POD** (evidence at protein level)	GAU72725.1	Fun_WK015_bin.7.fa_NODE_1292_length_14877_cov_9.784712	*Pichia fermentans*	35.028	177	110	4	5.31E-22	100
	GAU72725.1	Fun_WK038_bin.3.fa_NODE_431_length_17952_cov_14.525004	*Pichia membranifaciens*	35.593	177	109	4	1.29E-21	99.4
	GAU72725.1	Bac_WK001_bin.3.fa_NODE_45_length_107724_cov_66.965635	*Acetobacter papayae*	31.217	189	127	3	2.56E-20	95.5
	GAU72725.1	Fun_WK003_bin.15.fa_NODE_358_length_35075_cov_18.871473	*Brettanomyces bruxellensis*	32.418	182	117	3	2.60E-20	95.5
	GAU72725.1	Fun_WK052_bin.3.fa_NODE_214_length_62793_cov_78.228601	*Saccharomyces uvarum*	32.418	182	117	3	2.86E-20	95.5
	GAU72725.1	Fun_WK012_bin.10.fa_NODE_2698_length_3834_cov_6.683779	*Saccharomyces cerevisiae*	31.868	182	118	3	3.74E-20	95.1

Selection of the best tblastn hits of AMO and POD coding genes within WK MAGs. Only one hit for each species within the three protein groups was retained.

Supplementary References

1 Gertz, E. M., Yu, Y.-K., Agarwala, R., Schäffer, A. A. & Altschul, S. F. Composition-based statistics and translated nucleotide searches: Improving the TBLASTN module of BLAST. *BMC Biology* **4**, 41, doi:10.1186/1741-7007-4-41 (2006).

2 Wood, D. E., Lu, J. & Langmead, B. Improved metagenomic analysis with Kraken 2. *Genome Biology* **20**, 257, doi:10.1186/s13059-019-1891-0 (2019).

3 Lu, J., Breitwieser, F. P., Thielen, P. & Salzberg, S. L. Bracken: estimating species abundance in metagenomics data. *PeerJ Computer Science* **3**, e104 (2017).

4 Menzel, P., Ng, K. L. & Krogh, A. Fast and sensitive taxonomic classification for metagenomics with Kaiju. *Nature Communications* **7**, 11257, doi:10.1038/ncomms11257 (2016).

5 Beghini, F. *et al.* Integrating taxonomic, functional, and strain-level profiling of diverse microbial communities with bioBakery 3. *bioRxiv*, 2020.2011.2019.388223, doi:10.1101/2020.11.19.388223 (2020).

6 Verce, M., De Vuyst, L. & Weckx, S. Shotgun Metagenomics of a Water Kefir Fermentation Ecosystem Reveals a Novel Oenococcus Species. *Front Microbiol* **10**, 479, doi:10.3389/fmicb.2019.00479 (2019).

7 Olm, M. R. *et al.* inStrain profiles population microdiversity from metagenomic data and sensitively detects shared microbial strains. *Nature Biotechnology*, doi:10.1038/s41587-020-00797-0 (2021).

8 Oyserman, B. O. *et al.* Disentangling the genetic basis of rhizosphere microbiome assembly in tomato. *Nature Communications* **13**, 3228, doi:10.1038/s41467-022-30849-9 (2022).

9 Olm, M. R. *et al.* Robust variation in infant gut microbiome assembly across a spectrum of lifestyles. *Science* **376**, 1220-1223, doi:10.1126/science.abj2972 (2022).

10 Walsh, A. M. *et al.* Species classifier choice is a key consideration when analysing low-complexity food microbiome data. *Microbiome* **6**, 50, doi:10.1186/s40168-018-0437-0 (2018).

11 Ye, S. H., Siddle, K. J., Park, D. J. & Sabeti, P. C. Benchmarking Metagenomics Tools for Taxonomic Classification. *Cell* **178**, 779-794, doi:https://doi.org/10.1016/j.cell.2019.07.010 (2019).

12 Horisberger, M. Structure of the dextran of the Tibi grain. *Carbohydrate Research* **10**, 379-385, doi:https://doi.org/10.1016/S0008-6215(00)80897-6 (1969).

13 Fels, L., Jakob, F., Vogel, R. F. & Wefers, D. Structural characterization of the exopolysaccharides from water kefir. *Carbohydr Polym* **189**, 296-303, doi:10.1016/j.carbpol.2018.02.037 (2018).

14 Waldherr, F. W., Doll, V. M., Meissner, D. & Vogel, R. F. Identification and characterization of a glucan-producing enzyme from Lactobacillus hilgardii TMW 1.828 involved in granule formation of water kefir. *Food Microbiol* **27**, 672-678, doi:10.1016/j.fm.2010.03.013 (2010).

15 Bechtner, J., Wefers, D., Schmid, J., Vogel, R. F. & Jakob, F. Identification and comparison of two closely related dextransucrases released by water kefir borne Lactobacillus hordei TMW 1.1822 and Lactobacillus nagelii TMW 1.1827. *Microbiology (Reading)* **165**, 956-966, doi:10.1099/mic.0.000825 (2019).

16 Davidović, S. Z., Miljković, M. G., Antonović, D. G., Rajilić-Stojanović, M. D. & Dimitrijević-Branković, S. I. Water Kefir grain as a source of potent dextran producing lactic acid bacteria. *Hemijska industrija* **69**, 595-604 (2015).

17 van Hijum Sacha, A. F. T., Kralj, S., Ozimek Lukasz, K., Dijkhuizen, L. & van Geel-Schutten Ineke, G. H. Structure-Function Relationships of Glucansucrase and Fructansucrase Enzymes from Lactic Acid Bacteria. *Microbiology and Molecular Biology Reviews* **70**, 157-176, doi:10.1128/mmbr.70.1.157-176.2006 (2006).

18 Laureys, D., Van Jean, A., Dumont, J. & De Vuyst, L. Investigation of the instability and low water kefir grain growth during an industrial water kefir fermentation process. *Appl Microbiol Biotechnol* **101**, 2811-2819, doi:10.1007/s00253-016-8084-5 (2017).

19 Araújo, M. B. & Rozenfeld, A. The geographic scaling of biotic interactions. *Ecography* **37**, 406-415, doi:https://doi.org/10.1111/j.1600-0587.2013.00643.x (2014).
20 Morales-Castilla, I., Matias, M. G., Gravel, D. & Araújo, M. B. Inferring biotic interactions from proxies. *Trends in Ecology & Evolution* **30**, 347-356, doi:https://doi.org/10.1016/j.tree.2015.03.014 (2015).
21 Oren, A. & Garrity, G. Notification of changes in taxonomic opinion previously published outside the IJSEM. *International Journal of Systematic and Evolutionary Microbiology* **70**, 4061-4090, doi:https://doi.org/10.1099/ijsem.0.004245 (2020).
22 Panesar, P. S., Marwaha, S. S. & Kennedy, J. F. Zymomonas mobilis: an alternative ethanol producer. *Journal of Chemical Technology & Biotechnology* **81**, 623-635, doi:https://doi.org/10.1002/jctb.1448 (2006).
23 Laureys, D. & De Vuyst, L. The water kefir grain inoculum determines the characteristics of the resulting water kefir fermentation process. *J Appl Microbiol* **122**, 719-732, doi:10.1111/jam.13370 (2017).
24 Leech, J. *et al.* Fermented-Food Metagenomics Reveals Substrate-Associated Differences in Taxonomy and Health-Associated and Antibiotic Resistance Determinants. *mSystems* **5**, e00522-00520, doi:10.1128/mSystems.00522-20 (2020).
25 Patel, S. H. *et al.* A temporal view of the water kefir microbiota and flavour attributes. *Innovative Food Science & Emerging Technologies*, 103084, doi:https://doi.org/10.1016/j.ifset.2022.103084 (2022).
26 Marsh, A. J., O'Sullivan, O., Hill, C., Ross, R. P. & Cotter, P. D. Sequence-based analysis of the microbial composition of water kefir from multiple sources. *FEMS Microbiol Lett* **348**, 79-85, doi:10.1111/1574-6968.12248 (2013).
27 Kuhner, C. H. *et al.* Clostridium akagii sp. nov. and Clostridium acidisoli sp. nov.: acid-tolerant, N2-fixing clostridia isolated from acidic forest soil and litter. *International Journal of Systematic and Evolutionary Microbiology* **50**, 873-881, doi:https://doi.org/10.1099/00207713-50-2-873 (2000).
28 Kobayashi, H. *et al.* Reclassification of Clostridium diolis Biebl and Spröer 2003 as a later heterotypic synonym of Clostridium beijerinckii Donker 1926 (Approved Lists 1980) emend. Keis et al. 2001. *International Journal of Systematic and Evolutionary Microbiology* **70**, 2463-2466 (2020).
29 Biebl, H., Marten, S., Hippe, H. & Deckwer, W.-D. Glycerol conversion to 1,3-propanediol by newly isolated clostridia. *Applied Microbiology and Biotechnology* **36**, 592-597, doi:10.1007/BF00183234 (1992).
30 Biebl, H. & Spröer, C. Taxonomy of the Glycerol Fermenting Clostridia and Description of Clostridium diolis sp. nov. *Systematic and Applied Microbiology* **25**, 491-497, doi:https://doi.org/10.1078/07232020260517616 (2002).
31 Dürre, P. Physiology and Sporulation in Clostridium. *Microbiology Spectrum* **2**, 10.1128/microbiolspec.tbs-0010-2012, doi:10.1128/microbiolspec.tbs-0010-2012 (2014).
32 Hailegebreal, G. A review on Clostridium perfringens food poisoning. *Global Research Journal of Public Health and Epidemiology* **4**, 104-109 (2017).
33 Candel-Pérez, C., Ros-Berruezo, G. & Martínez-Graciá, C. A review of Clostridioides [Clostridium] difficile occurrence through the food chain. *Food Microbiology* **77**, 118-129, doi:https://doi.org/10.1016/j.fm.2018.08.012 (2019).
34 Anderson, N. M. *et al.* Food Safety Objective Approach for Controlling Clostridium botulinum Growth and Toxin Production in Commercially Sterile Foods. *Journal of Food Protection* **74**, 1956-1989, doi:https://doi.org/10.4315/0362-028X.JFP-11-082 (2011).
35 Gulitz, A., Stadie, J., Ehrmann, M. A., Ludwig, W. & Vogel, R. F. Comparative phylobiomic analysis of the bacterial community of water kefir by 16S rRNA gene amplicon sequencing and ARDRA analysis. *J Appl Microbiol* **114**, 1082-1091, doi:10.1111/jam.12124 (2013).

36 Laureys, D., Aerts, M., Vandamme, P. & De Vuyst, L. Oxygen and diverse nutrients influence the water kefir fermentation process. *Food Microbiol* **73**, 351-361, doi:10.1016/j.fm.2018.02.007 (2018).
37 Langendries, S. & Goormachtig, S. Paenibacillus polymyxa, a Jack of all trades. *Environmental Microbiology* **23**, 5659-5669, doi:https://doi.org/10.1111/1462-2920.15450 (2021).
38 Zega, A. NMR Methods for Identification of False Positives in Biochemical Screens. *Journal of Medicinal Chemistry* **60**, 9437-9447, doi:10.1021/acs.jmedchem.6b01520 (2017).
39 Malečková, M., Vrzal, T., Vaško, T., Olšovská, J. & Sobotníková, J. Natural Occurrence of Nitrite-Related Compounds in Malt and Beer. *Journal of Agricultural and Food Chemistry* **71**, 17321-17329, doi:10.1021/acs.jafc.3c05217 (2023).
40 Kazezoğlu, C. *et al.* Investigation of Urine Organic Acid Profile in Coronavirus Disease (COVID-19) Patients. *Clinical Laboratory* **70** (2024).
41 Yap, P. S. X. *et al.* Neonatal intensive care unit (NICU) exposures exert a sustained influence on the progression of gut microbiota and metabolome in the first year of life. *Scientific Reports* **11**, 1353, doi:10.1038/s41598-020-80278-1 (2021).
42 Bessonneau, V., Bojko, B. & Pawliszyn, J. Analysis of Human Saliva Metabolome by Direct Immersion Solid-Phase Microextraction LC and Benchtop Orbitrap MS. *Bioanalysis* **5**, 783-792, doi:10.4155/bio.13.35 (2013).
43 Arp, D. J., Sayavedra-Soto, L. A. & Hommes, N. G. Molecular biology and biochemistry of ammonia oxidation by Nitrosomonas europaea. *Archives of Microbiology* **178**, 250-255, doi:10.1007/s00203-002-0452-0 (2002).
44 Ono, Y., Enokiya, A., Masuko, D., Shoji, K. & Yamanaka, T. Pyruvic Oxime Dioxygenase from the Heterotrophic Nitrifier Alcaligenes faecalis: Purification, and Molecular and Enzymatic Properties. *Plant and Cell Physiology* **40**, 47-52, doi:10.1093/oxfordjournals.pcp.a029473 (1999).
45 pyru, Tsujino, S., Uematsu, C., Dohra, H. & Fujiwara, T. Pyruvic oxime dioxygenase from heterotrophic nitrifier Alcaligenes faecalis is a nonheme Fe(II)-dependent enzyme homologous to class II aldolase. *Scientific Reports* **7**, 39991, doi:10.1038/srep39991 (2017).
46 Tsujino, S. *et al.* Phylogenetic diversity, distribution, and gene structure of the pyruvic oxime dioxygenase involved in heterotrophic nitrification. *Antonie van Leeuwenhoek* **116**, 1037-1055, doi:10.1007/s10482-023-01862-9 (2023).
47 Stadie, J. Metabolic activity and symbiotic interaction of bacteria and yeasts in water kefir. (2013).
48 Stadie, J., Gulitz, A., Ehrmann, M. A. & Vogel, R. F. Metabolic activity and symbiotic interactions of lactic acid bacteria and yeasts isolated from water kefir. *Food Microbiol* **35**, 92-98, doi:10.1016/j.fm.2013.03.009 (2013).
49 Hariyanto, Sarno, R. & Wijaya, D. R. in *2017 11th International Conference on Information & Communication Technology and System (ICTS).* 241-246.
50 Tharmakulasingam, M., Topal, C., Fernando, A. & Ragione, R. L. in *Proceedings of the 2019 6th International Conference on Biomedical and Bioinformatics Engineering* 118–124 (Association for Computing Machinery, Shanghai, China, 2020).
51 Magalhaes, K. T., de, M. P. G. V., Dias, D. R. & Schwan, R. F. Microbial communities and chemical changes during fermentation of sugary Brazilian kefir. *World J Microbiol Biotechnol* **26**, 1241-1250, doi:10.1007/s11274-009-0294-x (2010).
52 Arrieta-Echeverri, M. C. *et al.* Multi-omics characterization of the microbial populations and chemical space composition of a water kefir fermentation. *Frontiers in Molecular Biosciences* **10**, doi:10.3389/fmolb.2023.1223863 (2023).
53 Luang-In, V. *et al.* Microbial strains and bioactive exopolysaccharide producers from Thai water kefir. *Microbiology and Biotechnology Letters* **46**, 403-415 (2018).

54 Laureys, D., Cnockaert, M., De Vuyst, L. & Vandamme, P. Bifidobacterium aquikefiri sp. nov., isolated from water kefir. *Int J Syst Evol Microbiol* **66**, 1281-1286, doi:10.1099/ijsem.0.000877 (2016).
55 Laureys, D. & De Vuyst, L. Microbial species diversity, community dynamics, and metabolite kinetics of water kefir fermentation. *Appl Environ Microbiol* **80**, 2564-2572, doi:10.1128/AEM.03978-13 (2014).
56 Laureys, D., Aerts, M., Vandamme, P. & De Vuyst, L. The Buffer Capacity and Calcium Concentration of Water Influence the Microbial Species Diversity, Grain Growth, and Metabolite Production During Water Kefir Fermentation. *Front Microbiol* **10**, 2876, doi:10.3389/fmicb.2019.02876 (2019).
57 Eckel, V. P. L., Ziegler, L. M., Vogel, R. F. & Ehrmann, M. Bifidobacterium tibiigranuli sp. nov. isolated from homemade water kefir. *Int J Syst Evol Microbiol* **70**, 1562-1570, doi:10.1099/ijsem.0.003936 (2020).
58 Zhu, H.-Y. *et al.* Pichia kurtzmaniana f.a. sp. nov., with the transfer of eight Candida species to Pichia. *International Journal of Systematic and Evolutionary Microbiology* **74**, doi:https://doi.org/10.1099/ijsem.0.006306 (2024).
59 Martínez-Torres, A., Gutiérrez-Ambrocio, S., Heredia-del-Orbe, P., Villa-Tanaca, L. & Hernández-Rodríguez, C. Inferring the role of microorganisms in water kefir fermentations. *International Journal of Food Science & Technology* **52**, 559-571, doi:https://doi.org/10.1111/ijfs.13312 (2017).
60 da C. P. Miguel, M. G., Cardoso, P. G., Magalhães, K. T. & Schwan, R. F. Profile of microbial communities present in tibico (sugary kefir) grains from different Brazilian States. *World Journal of Microbiology and Biotechnology* **27**, 1875-1884, doi:10.1007/s11274-010-0646-6 (2011).
61 Hundschell, C. S., Braun, A., Wefers, D., Vogel, R. F. & Jakob, F. Size-Dependent Variability in Flow and Viscoelastic Behavior of Levan Produced by Gluconobacter albidus TMW 2.1191. *Foods* **9**, doi:10.3390/foods9020192 (2020).
62 Zannini, E. *et al.* Influence of Substrate on the Fermentation Characteristics and Culture-Dependent Microbial Composition of Water Kefir. *Fermentation* **9** (2023).
63 Rios, D. L. *et al.* Comparative metatranscriptome analysis of Brazilian milk and water kefir beverages. *International Microbiology*, doi:10.1007/s10123-023-00431-4 (2023).
64 Gulitz, A., Stadie, J., Wenning, M., Ehrmann, M. A. & Vogel, R. F. The microbial diversity of water kefir. *Int J Food Microbiol* **151**, 284-288, doi:10.1016/j.ijfoodmicro.2011.09.016 (2011).
65 Zavala, L. *et al.* Selected Lactobacillus strains isolated from sugary and milk kefir reduce Salmonella infection of epithelial cells in vitro. *Benef Microbes* **7**, 585-595, doi:10.3920/BM2015.0196 (2016).
66 Zanirati, D. F. *et al.* Selection of lactic acid bacteria from Brazilian kefir grains for potential use as starter or probiotic cultures. *Anaerobe* **32**, 70-76, doi:10.1016/j.anaerobe.2014.12.007 (2015).
67 Cao, C. *et al.* Assessment of the microbial diversity of Chinese Tianshan tibicos by single molecule, real-time sequencing technology. *Food Sci Biotechnol* **28**, 139-145, doi:10.1007/s10068-018-0460-8 (2019).
68 Hsieh, H. H., Wang, S. Y., Chen, T. L., Huang, Y. L. & Chen, M. J. Effects of cow's and goat's milk as fermentation media on the microbial ecology of sugary kefir grains. *Int J Food Microbiol* **157**, 73-81, doi:10.1016/j.ijfoodmicro.2012.04.014 (2012).
69 Xu, D. *et al.* Lifestyle of Lactobacillus hordei isolated from water kefir based on genomic, proteomic and physiological characterization. *Int J Food Microbiol* **290**, 141-149, doi:10.1016/j.ijfoodmicro.2018.10.004 (2019).
70 Koh, W. Y., Utra, U., Ahmad, R., Rather, I. A. & Park, Y. H. Evaluation of probiotic potential and anti-hyperglycemic properties of a novel Lactobacillus strain isolated from water kefir grains. *Food Sci Biotechnol* **27**, 1369-1376, doi:10.1007/s10068-018-0360-y (2018).

71 Brandão, P. R., Crespo, M. T. B. & Nascimento, F. X. Phylogenomic and comparative analyses support the reclassification of several Komagataeibacter species as novel members of the Novacetimonas gen. nov. and bring new insights into the evolution of cellulose synthase genes. *International Journal of Systematic and Evolutionary Microbiology* **72**, 005252 (2022).

72 Verce, M., De Vuyst, L. & Weckx, S. The metagenome-assembled genome of Candidatus Oenococcus aquikefiri from water kefir represents the species Oenococcus sicerae. *Food Microbiol* **88**, 103402, doi:10.1016/j.fm.2019.103402 (2020).

73 Diosma, G., Romanin, D. E., Rey-Burusco, M. F., Londero, A. & Garrote, G. L. Yeasts from kefir grains: isolation, identification, and probiotic characterization. *World J Microbiol Biotechnol* **30**, 43-53, doi:10.1007/s11274-013-1419-9 (2014).

74 Romero-Luna, H. E., Hernandez-Sanchez, H., Ribas-Aparicio, R. M., Cauich-Sanchez, P. I. & Davila-Ortiz, G. Evaluation of the Probiotic Potential of Saccharomyces cerevisiae Strain (C41) Isolated from Tibicos by In Vitro Studies. *Probiotics Antimicrob Proteins* **11**, 794-800, doi:10.1007/s12602-018-9471-2 (2019).

75 Picozzi, C., Clagnan, E., Musatti, A., Rollini, M. & Brusetti, L. Characterization of Two Zymomonas mobilis Wild Strains and Analysis of Populations Dynamics during Their Leavening of Bread-like Doughs. *Foods* **11** (2022). <https://mdpi-res.com/d_attachment/foods/foods-11-02768/article_deploy/foods-11-02768-v2.pdf?version=1662715566>.
